# Single-colony sequencing reveals microbe-by-microbiome phylosymbiosis between the cyanobacterium *Microcystis* and its associated bacteria

**DOI:** 10.1186/s40168-021-01140-8

**Published:** 2021-09-27

**Authors:** Olga M. Pérez-Carrascal, Nicolas Tromas, Yves Terrat, Elisa Moreno, Alessandra Giani, Laisa Corrêa Braga Marques, Nathalie Fortin, B. Jesse Shapiro

**Affiliations:** 1 Département de Sciences Biologiques, Université́ de Montréal, Montréal, Québec Canada; 2 Federal University of Minas Gerais, Belo Horizonte, Minas Gerais Brazil; 3 National Research Council of Canada, Montreal, Québec Canada; 4 Department of Microbiology & Immunology, McGill University, Montreal, Québec Canada; 5 McGill Genome Centre, McGill University, Montreal, Québec Canada

**Keywords:** *Microcystis*, Cyanobacteria, Phylosymbiosis, Co-phylogeny, Microbiome, Phycosphere

## Abstract

**Background:**

Cyanobacteria from the genus *Microcystis* can form large mucilaginous colonies with attached heterotrophic bacteria—their microbiome. However, the nature of the relationship between *Microcystis* and its microbiome remains unclear. Is it a long-term, evolutionarily stable association? Which partners benefit? Here we report the genomic diversity of 109 individual *Microcystis* colonies—including cyanobacteria and associated bacterial genomes—isolated *in situ* and without culture from Lake Champlain, Canada and Pampulha Reservoir, Brazil.

**Results:**

We identified 14 distinct *Microcystis* genotypes from Canada, of which only two have been previously reported, and four genotypes specific to Brazil. *Microcystis* genetic diversity was much greater between than within colonies, consistent with colony growth by clonal expansion rather than aggregation of *Microcystis* cells. We also identified 72 bacterial species in the microbiome. Each *Microcystis* genotype had a distinct microbiome composition, and more closely related genotypes had more similar microbiomes. This pattern of phylosymbiosis could be explained by co-phylogeny in only two out of the nine most prevalent associated bacterial genera, *Roseomonas* and *Rhodobacter*. These phylogenetically associated genera could enrich the metabolic repertoire of *Microcystis,* for example by encoding the biosynthesis of complementary carotenoid molecules*.* In contrast, other colony-associated bacteria showed weaker signals of co-phylogeny, but stronger evidence of horizontal gene transfer with *Microcystis*. These observations suggest that acquired genes are more likely to be retained in both partners (*Microcystis* and members of its microbiome) when they are loosely associated, whereas one gene copy is sufficient when the association is physically tight and evolutionarily long-lasting.

**Conclusions:**

We have introduced a method for culture-free isolation of single colonies from nature followed by metagenomic sequencing, which could be applied to other types of microbes. Together, our results expand the known genetic diversity of both *Microcystis* and its microbiome in natural settings, and support their long-term, specific, and potentially beneficial associations.

Video Abstract

**Supplementary Information:**

The online version contains supplementary material available at 10.1186/s40168-021-01140-8.

## Background

Cyanobacteria occur naturally in aquatic ecosystems, often multiplying into harmful blooms and producing a diversity of toxins, which can cause severe human illness [1]. Many cyanobacteria and eukaryotic algae grow in mucilaginous colonies surrounded by a zone, called the phycosphere, rich in cell exudates, where metabolites are exchanged between numerous microorganisms [2, 3]. In this microhabitat, the interactions between cyanobacteria and associated bacteria (AB) might include mutualism (with all partners benefitting), competition (with all partners competing for resources), antagonism (inhibiting one of the partners), commensalism (with one partner benefitting) and parasitism (with one partner benefitting at the expense of the other) [3–5]. However, the drivers shaping these associations are largely unknown. In some cases, AB may enhance algal or cyanobacterial growth [6, 7], aiding in phosphorus acquisition in *Trichodesmium* for example [8, 9]. Understanding the contributions of AB to cyanobacterial growth and toxin production has implications for our ability to predict and control harmful blooms.

*Microcystis* is a globally distributed, often toxigenic bloom-forming freshwater cyanobacterium, which forms macroscopic mucilaginous colonies. These colonies offer a nutrient-rich habitat for other bacteria, while also providing physical protection against grazers [10–12]. The *Microcystis* colony microbiome is distinct from the surrounding lake bacterial community, enriched in *Proteobacteria* and depleted in *Actinobacteria* [13, 14]. The microbiome composition has been associated with temperature, seasonality, biogeography, *Microcystis* morphology and density [13, 15–17]. Lab experiments show the potential for AB to influence *Microcystis* growth and colony formation [18–21]. Yet it remains unclear whether such interactions are relevant in natural settings, and if they are the product of long-term associations over evolutionary time. *Microcystis* interacts with eukaryotes through competition with algae and predation by zooplankton [22], but eukaryotes are not known to be physically associated with *Microcystis* colonies. In contrast, sequencing and microscopy have revealed physical associations between *Microcystis* and smaller bacterial cells [23]. The *in situ* isolation of *Microcystis* from lakes offers an opportunity to further elucidate the associations between *Microcystis* and specific members of its microbiome.

Phylosymbiosis, a pattern in which microbiome composition mirrors the host phylogeny, provides a useful concept for the study of host–microbiome associations and interactions [24]. Phylosymbiosis could arise from some combination of (1) vertical transmission of the microbiome from parent to offspring, resulting in co-speciation and shared phylogenetic patterns (co-phylogeny), (2) horizontal transmission of the microbiome, but with strong matching between hosts and microbiomes at each generation, and (3) co-evolution, in which hosts and microbiomes mutually impose selective pressures and adapt to each other. Distinguishing the relative importance of these three possibilities can be challenging, but in all cases, the associations between hosts and microbiomes must be significantly non-random in order for phylosymbiosis to be supported. Phylosymbiosis is typically studied between plant or animal hosts and their microbiomes [25–27] but *Microcystis* could also be considered a host, since it constructs the mucilage environment—although it is unclear to what extent it selects its AB or *vice versa*. *Microcystis* colonies are more open to the outside environment compared with mammalian guts, for example. Consequently, they might behave more like coral mucus [27] or other animal surfaces which seem to show weaker phylosymbiosis than guts [28]. The enclosed nature of animal guts reduces dispersal of microbiomes and favours vertical transmission, potentially leading to co-phylogeny without the need to invoke co-evolution [29]. In contrast, metagenomic sequencing suggests *Microcystis* and its microbiome are globally distributed [16], making it unlikely that phylosymbiosis could arise due to common biogeography of *Microcystis* and its microbiome. On the other hand, *Microcystis* may be geographically structured on shorter evolutionary time scales, due to local adaptation or clonal expansions, and *Microcystis* genotypes might have distinct phenotypic characteristics that could select for distinct microbiomes [30, 31]. Phylosymbiosis studies to date are biased toward the gut relative to external host compartments [24]. *Microcystis* colonies thus provide an alternative model of a more external microbiome in which to study “microbe-by-microbiome” phylosymbiosis.

Previous studies of the *Microcystis* microbiome have used either culture-independent metagenomics from lakes, a bulk biomass collection method which cannot resolve fine-scale spatial interaction within colonies (*e.g.*, [16]), or culture-based studies of *Microcystis* isolates, which have found host–microbiome divergence according to phosphorous gradients and taxonomy [32], but may not be representative of the natural diversity of *Microcystis* or AB as they occur in nature. To combine the strengths of both these approaches, we developed a simple method for isolating individual *Microcystis* colonies directly from lakes, followed by DNA extraction and sequencing without a culture step [31]. Here we applied this method to 109 individual colonies from Lake Champlain, Canada and Pampulha Reservoir, Brazil, yielding 109 *Microcystis* genomes and 391 AB genomes. This genomic dataset allowed us (i) to quantify the *Microcystis* genotype diversity in lakes over time; (ii) to identify associations between *Microcystis* genotypes and microbiome composition and assess the evidence for phylosymbiosis; and finally, (iii) to test for possible horizontal gene transfer (HGT) between *Microcystis* and members of its microbiome.

Our findings reveal an expanded *Microcystis* genotypic diversity, and a *Microcystis* colony microbiome shaped by the host genotype, resulting in a significant signature of phylosymbiosis. We inferred co-speciation of *Microcystis* with two of the most prevalent genera in its microbiome (*Rhodobacter* and *Roseomonas*) suggesting evolutionarily stable associations. We also inferred HGT events among *Microcystis* and its microbiome, mainly involving lower-fidelity partners than *Rhodobacter* and *Roseomonas*. Overall, our results suggest ecologically and evolutionarily stable associations between *Microcystis* and members of its microbiome.

## Results

### Genotypic diversity of *Microcystis* colonies in Lake Champlain and Pampulha Reservoir

**Fig. 1:**
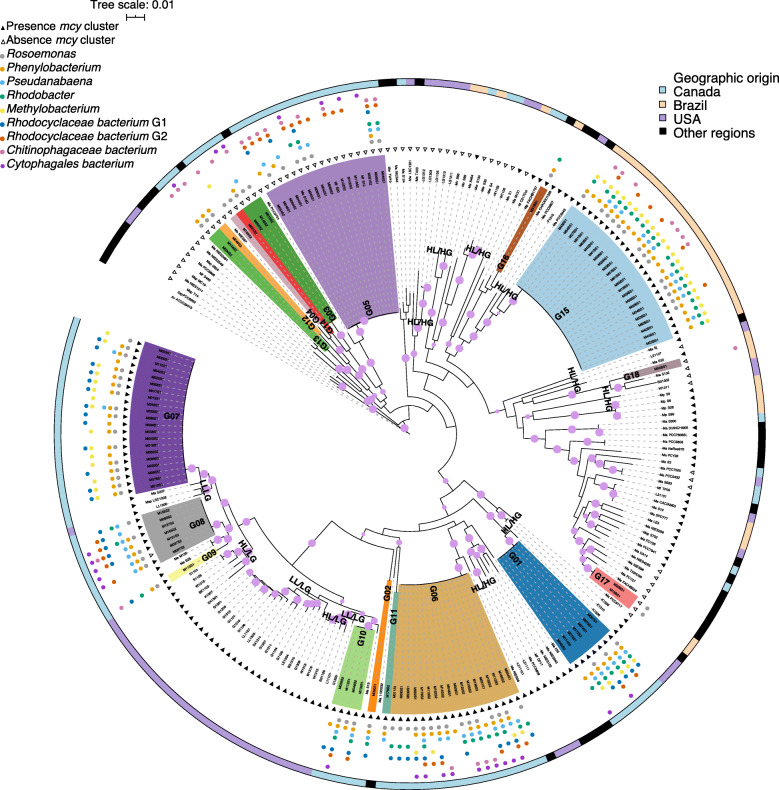
Maximum likelihood phylogenetic tree of 109 *Microcystis* colony genomes and previously sequenced reference genomes. *Microcystis* genomes were classified in 18 genotypes based on Average Nucleotide Identity (ANI) greater or equal to 99%. A core genome was inferred based on 109 *Microcystis* genomes and 122 *Microcystis* reference genomes downloaded from NCBI. The alignment of the 115 core genes (68,145 bp in total after excluding positions with gaps) was used to infer the Maximum Likelihood phylogeny. The tree was rooted using two cyanobacteria (*Anabaena variabilis* ATCC29413 and *Synechocystis* sp. PCC6803) as outgroups. The clades highlighted in different colours indicate *Microcystis* genotypes (G01 to G18) from this study; uncoloured clades are other reference genomes from the literature. The purple circles on the tree branches indicate bootstrap values greater or equal to 70%. The empty and filled triangles around the tree indicate absence and presence of the *mcy* cluster, respectively. The small coloured and filled dots indicate the most prevalent AB genera related to each *Microcystis* genome. The outermost circle indicates the geographic origin of the *Microcystis* genomes. Several reference genomes of *Microcystis* genotypes recovered from environments with high and low phosphorus are indicated as LL/LG (Low Phosphorus Lake/Low Phosphorus genotype), HL/LG (High-Phosphorus Lake/Low-Phosphorus Genotype) and HL/HG (High-Phosphorus Lake/High-Phosphorus Genotype). For details about the names of the strains, please refer to Supplementary Table 2

To study the relationship between *Microcystis* and its AB in natural settings, we sequenced 109 individual *Microcystis* colonies from 16 lake samples (82 colonies from Lake Champlain, Quebec, Canada and 27 from Pampulha Reservoir, Minas Gerais, Brazil; Supplementary Table 1). *Microcystis* genomes were assembled and binned separately from AB genomes (Methods), which we will describe below. *Microcystis* genomes recovered from the colonies have more than 90% completeness and less than 10% redundance based on a set of 139 single-copy core genes [33], except for M04BS1, which has 82.7% completeness (Supplementary Table 1), with an average genome size of 4.76 Mb. Consistent with our previous study of *Microcystis* isolate genomes [31], nearly all the 109 *Microcystis* genomes and the 122 reference genomes (average genome size of 4.85 Mb) share ≥ 95% average nucleotide identity (ANI), except for 7 out of 26,565 genome pairs which had ANI between 94.4 and 94.5%. The 95% ANI threshold is typically used to define bacterial species, but we previously found significant phylogenetic substructure above 95%, coherent with multiple species or ecotypes within *Microcystis* [31]. In agreement with such fine genetic structure within our sampled colonies, we identified 18 monophyletic, closely related genotypes of *Microcystis* (*i.e.,* ≥ 99.9% ANI and phylogenetic pairwise distances lower than 0.0005; Supplementary Table 2 and Supplementary Fig. 1). These genotypes (highlighted clades in Fig. 1) are nested within the phylogeny of 122 isolate genomes previously sampled from North America, Brazil, and worldwide. However, only two genotypes (G05 and G10) have been reported in culture previously, possibly due to our fine-grained definition of genotypes (≥ 99% ANI) combined with undersampling of natural diversity in culture collections [34]. Consistent with previously observed biogeographic patterns between North and South America [31], we found 14 genotypes unique to Lake Champlain, and four unique to Pampulha, with no genotypes found in both locations.

Two features of interest that might distinguish *Microcystis* clades are toxigenicity (the presence of the *mcy* operon encoding microcystin biosynthesis [35]) and adaptation to low nutrient conditions. *Microcystis* is generally thought to be adapted to high nutrient conditions, since it often blooms in eutrophic waters such as Champlain and Pampulha (Supplementary Table 3). However, a recent sampling of Michigan lakes identified *Microcystis* isolates adapted to low-phosphorus (low-phosphorus genotypes, LG), which occur in both high- and low-phosphorus lakes [32]. Genotypes G07, G08, G09 and G10 from Lake Champlain are nested within the LG clade with high bootstrap support (Fig. 1), indicating that low-phosphorus-adapted genotypes also occur in high-phosphorus lakes. Contrary to the single LG clade, high-phosphorus genotypes (HG) are broadly distributed across the phylogenetic tree and are recovered from multiple geographic locations. We observed that most of the genomes within the LG clade (66 out of 67) encode the *mcy* gene cluster (Fig. 1). In contrast, *mcy* was more unevenly distributed across HG genomes, possibly due to multiple gene gain/loss events. However, closely related genotypes tend to have identical *mcy* gene profiles, suggesting that putative gain/loss events occurred mainly in deep internal branches of the phylogeny.

### Lower *Microcystis* diversity within than between colonies of the same genotype suggests clonal colony formation

**Fig. 2:**
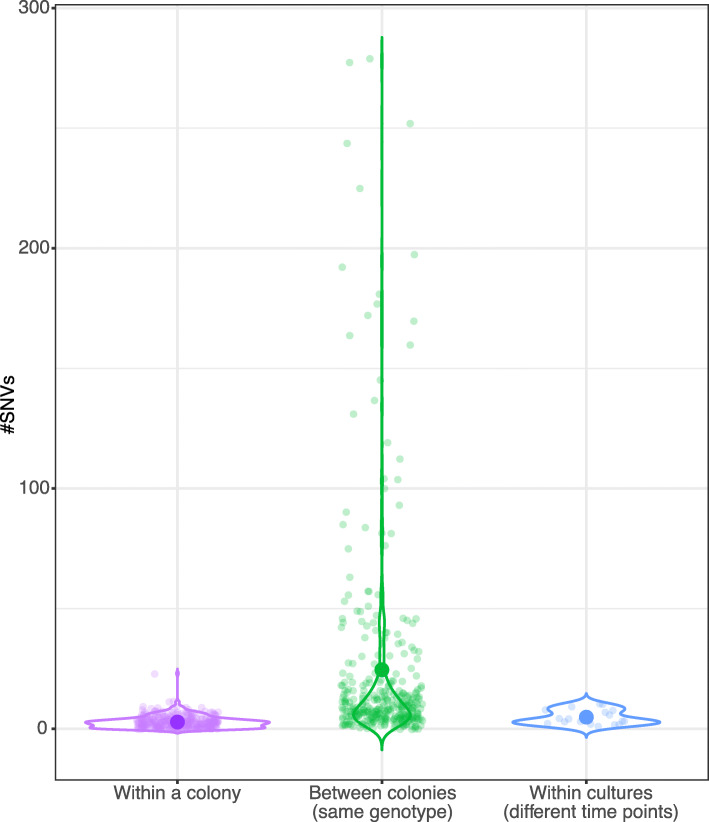
Greater genetic diversity between than within *Microcystis* colonies. The number of single-nucleotide variants (SNVs) within and between *Microcystis* colonies of the same genotype are shown, compared with SNVs that accumulated over ~ 6 years of *Microcystis* culture in the laboratory (Methods). Large points show mean values. The data points represent the SNVs in a *Microcystis* genome within a colony (purple dots), SNVs between *Microcystis* genomes from the same genotypic clade (green dots), and SNVs between *Microcystis* genomes from the same *Microcystis* culture at two different time points (blue dots). Within colonies, the comparison was done by mapping the reads from a colony against its corresponding assembled *Microcystis* genome. Nucleotide sites that differed between some fraction of the reads and the assembled reference genome were considered as SNVs within a colony (the lowest allele frequency found within a colony was of 0.14%)

A previous study of Michigan lakes supported clonal colony formation (by cell division) in isolates from high-phosphorus lakes, but suggested a preponderance of nonclonal colonies (by agglomeration of distantly related cell) in low-phosphorus lakes [32]. To further explore whether *Microcystis* colonies emerge from a single clonal cell or from an aggregation of either genetically diverse, we compared genetic diversity within and between colonies of the same genotype. Note that *Microcystis* genotypes are defined by ANI values greater than 99.9% and phylogenetic pairwise distances lower than 0.0005, and are thus not entirely isogenic. Within a colony, the number of single-nucleotide variants (SNVs) was significantly lower (mean of 3 and median of 2 SNVs) than between colonies (mean of 25 and median of 9 SNVs) of the same genotype (two-tailed Wilcoxon Rank Sum Test, *P* < 0.05; twelve outliers with more than 300 variants between colonies were excluded, making the test conservative) (Fig. 2 and Supplementary Table 4). These outliers were found in colonies within the genotypes G05, G06, G08 and G13. To put these results in context, we compared isolate genomes from 19 laboratory cultures of *Microcystis* sequenced in a previous study [31]. *Microcystis* accumulated an average of 5 SNVs after ~ 6 years of culture, with slightly more variation than observed within a colony but still ~ 5 times less than observed between colonies of the same genotype (two-tailed Wilcoxon Rank Sum Test, *P* < 0.05). Overall, these results are consistent with clonal expansion of *Microcystis* genomes within a colony—at least under the sampled environmental conditions in Lake Champlain and Pampulha Reservoir.

### Evidence for phylosymbiosis between *Microcystis* and its microbiome

Having characterized the genetic diversity of *Microcystis*, we turned our attention to the colony-associated bacteria (AB). We recovered a total of 391 non-*Microcystis* metagenome-assembled genomes (MAGs, with completeness ≥ 70 and contamination < 10%) from the 109 colonies (Supplementary Table 1 and 5), taxonomically classified into 72 putative species (ANI > 95%) and 37 genera. No MAGs were classified as eukaryotes, and only 0.21% of the contigs in the MAGs with completeness < 70 and contamination > 10% were assigned a likely eukaryotic origin. We thus tentatively conclude that eukaryotes are rare or undetectable in the *Microcystis* microbiome, as defined here. Only five AB species were shared among colonies from Canada and Brazil: *Pseudanabaena* sp. A06, *Methylobacterium* sp. A30, *Roseomonas* sp. A21, *Burkholderia* sp. A55 (a likely contaminant, as discussed below) and *Gemmatimonas* sp. A63 (Supplementary Fig. 2). Because certain low-abundance AB might be present in a colony but fail to assemble, we mapped reads from each colony to a database of all MAGs and estimated AB genome coverages. Using this approach, we found that each colony contained an average of six AB (genome coverage greater or equal to 1×), with a range of 0 to 15 (Supplementary Fig. 3). We found no strict “core” of AB present in all colonies, either at the species or genus level. However, several genera were quite prevalent. These include *Phenylobacterium* (present in 73.40% of colonies), *Roseomonas* (70.64%), *Pseudanabaena* (43.12%), *Rhodobacter* (46.79%), *Methylobacterium* (44.04%), *Rhodocyclaceae* G1 (unclassified genus) (39.45%), *Rhodocyclaceae* G2 (unclassified genus) (31.19%), *Chitinophagaceae* (unclassified genus) (26.60%), and *Cytophagales* (unclassified genus) (22.94%). It is possible that a core *Microcystis* microbiome does exist, but includes rare AB that were not assembled into MAGs. To test this possibility, we used two alternative assembly-independent metagenomic read mapping approaches to define the microbiome: Kaiju and MIDAS (Methods). We could not find a core microbiome based on the reference MIDAS database, which contains more than 30,000 reference genomes classified into 5952 species. With Kaiju, which is based on NCBI Reference Sequences, we identified between 0 and 78 core species depending on the read count cutoff (Supplementary Fig. 4 and Supplementary Table 6). However, the core microbiome detected by Kaiju is likely due to misclassification of reads at the species level, which poorly matches the species classification based on MIDAS. For example, the inferred core species supported by at least 1000 reads by Kaiju (still less than 1× coverage of a typical bacterial genome) were *Pseudomonas aeruginosa* and an *Acidobacteria* species, which we consider likely contaminants. We therefore conclude that, at the resolution of reasonably deep metagenomic sequencing, a strict core microbiome does not exist.

**Fig. 3:**
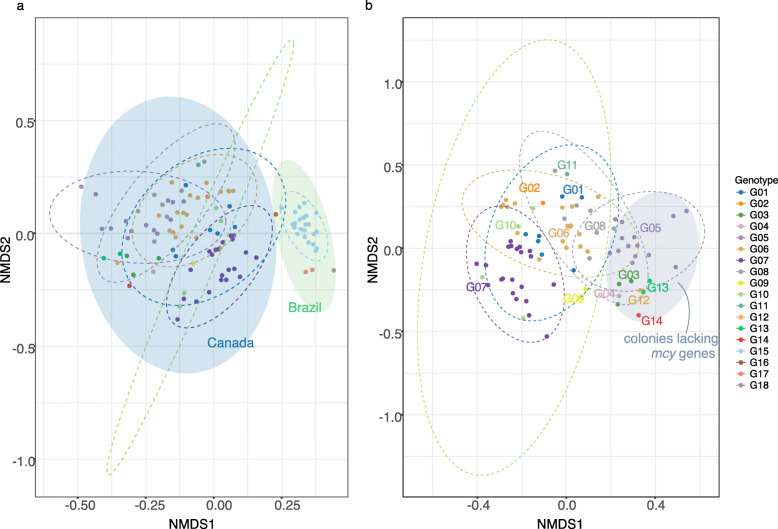
*Microcystis* genotypes have distinct microbiomes. Nonmetric multidimensional scaling (NMDS) plots are based on the coverage of the non-*Microcystis* metagenome-assembled genomes (MAGs) per colony (Bray–Curtis distance). **a** All samples, including those from Pampulha, Brazil and Lake Champlain, Canada. Ellipses show 95% confidence intervals of **b** samples from Lake Champlain only (stress = 0.225). The grey-shaded ellipse shows *Microcystis* colonies that do not encode the *mcy* cluster for microcystin toxin production

To assess the evidence for phylosymbiosis, we first asked if different *Microcystis* genotypes had distinct colony microbiomes. The phylogeny illustrates how certain *Microcystis* genotypes appear to be preferentially associated with particular AB (Fig. 1). For example, *Phenylobacterium* and *Methylobacterium* were present in all sampled colonies of genotype G15, while *Rhodobacter* and *Phenylobacterium* occur in all colonies of genotype G01. These anecdotal patterns are borne out in statistical analyses of colony community structure, which show that *Microcystis* genotypes have significantly distinct microbiomes relative to the null model of random assignment of AB across genotypes (Fig. 3a). The genotype explains more variation in community structure (PERMANOVA on Bray–Curtis distances, *R*^*2*^ = 38.7%, *P* < 0.01; Supplementary Table 7) than any other measured variable including pH (*R*^*2*^ ≤ 5%) or temperature at the sampling site (*R*^*2*^ ≤ 5%), presence of microcystin (*mcy*) genes in the genotype (*R*^*2*^ ≤ 5%), or location of sampling (*R*^*2*^ = 11.8%). Genotype was still the best explanatory variable when the analysis was performed on Lake Champlain samples only to exclude the effects of geography (Fig. 3b, PERMANOVA, *R*^*2*^ = 30.9%, *P* = 0.001).

A key piece of evidence for phylosymbiosis is not only for microbiomes to differ among host lineages, but for microbiome composition to change proportionally to host phylogeny. To test this, we converted the *Microcystis* host phylogeny into a distance matrix, which we correlated with the colony microbiome Bray–Curtis dissimilarity matrix. Consistent with phylosymbiosis, we found that variation in microbiome composition was correlated with the host phylogeny according to a Mantel test (*r* = 0.5, *P* = 0.001), and further confirmed with Procrustean superimposition (*r* = 0.6, *P* = 0.001) [36]. Similar evidence for phylosymbiosis was obtained using Kaiju or an expanded MIDAS reference genome database (Supplementary Table 8). While these alternative approaches yielded significant Mantel and Procrustes tests, the correlation coefficients were slightly lower than obtained with our set of 72 MAGs. This suggests that the alternative methods, which may be more sensitive to rare, unassembled taxa, likely add noise rather than strengthen the signal of phylosymbiosis. Taken together, these results provide strong support for phylosymbiosis in *Microcystis* colonies.

### *Microcystis* genotype abundances vary over time in Lake Champlain and are correlated with prevalent members of the microbiome

Toxigenic and non-toxigenic genotypes of *Microcystis* are known to change in relative abundance within lakes over time [34, 37, 38]. More generally, to what extent different genotypes of *Microcystis* vary over time, along with their colony-associated bacteria, is less well studied. To investigate the *Microcystis* genotype diversity in metagenomes from Lake Champlain, we used a database of 15 single-copy taxonomic marker genes, which are mostly involved in translation, and ribosomal structure and biogenesis (Supplementary Table 9) [39]. This database was used to distinguish among the 14 *Microcystis* genotypes identified from colony sequencing (Methods), and to estimate their relative abundance in 72 metagenomes sampled from filtered lake water during the summer months of 2006 to 2018 (Supplementary Fig. 5). It is possible that these 14 genotypes do not represent the total genotypic diversity of *Microcystis* occurring in the lake. However, mapping metagenomic reads from the lake to these genotypes with a 99% sequence identity threshold allowed us to recover 93.5% of *Microcystis* reads. *Microcystis* reads were defined at > 96% sequence identity to the *Microcystis* reference genome M083S1 (Methods). Therefore, the 14 genotypes are representative of the vast majority of *Microcystis* diversity captured in lake metagenomes.

Using a distance-based redundancy analysis (dbRDA), we estimated the effects of total phosphorous, total nitrogen, dissolved phosphorous, dissolved nitrogen, mean temperature and time (years, months and season) on the *Microcystis* genotype community composition in the 42 Lake Champlain metagenomes with complete metadata, and with *Microcystis* genome coverage greater or equal to 1×. *Microcystis* genotype diversity was best explained by yearly temporal variation (dbRDA, *R*^*2*^ = 51.1%, *P* < 0.01; PERMANOVA, *R*^*2*^ = 47.9%, *P* < 0.01; Supplementary Fig. 6). Years did not differ significantly in their dispersion (PERMDISP *P* > 0.05; Supplementary Table 7). Environmental variables such as nitrogen and phosphorus did not have a significant effect on the community composition. In a shorter time series (April to November of 1 year) in Pampulha, a more diverse community of four *Microcystis* genotypes eventually became dominated by one genotype (G15) encoding the *mcy* toxin biosynthesis gene cluster (Supplementary Fig. 7). However, more extensive sampling is required to estimate the effect of other environmental variables (*e.g*., phosphorus) on the community composition in Brazil.

Similarly to *Microcystis* genotypes*,* the composition of AB in Lake Champlain also varied significantly across years (dbRDA, *R*^*2*^ = 44.3%, *P* < 0.01; PERMANOVA, *R*^*2*^ = 43.5%, *P* < 0.01; Supplementary Fig. 8). We asked if the presence of dominant *Microcystis* genotypes could explain the variation in the AB community composition. A significant effect of the genotype was observed using PERMANOVA (*R*^*2*^ = 31.5%, *P* < 0.01), but not using dbRDA (*R*^*2*^ = 0.012, *P* > 0.05). Years and *Microcystis* genotypes were the best explanatory variables for AB composition; however, their dispersions were significantly different (*P* < 0.01) making the PERMANOVA results difficult to interpret. In addition, the AB community sampled from metagenomes includes both free-living and colony-attached AB, possibly adding noise to any signal of *Microcystis* genotypes selecting for specific AB within colonies. While these results are statistically somewhat ambiguous, they are generally consistent with phylosymbiosis between *Microcystis* and its microbiome.

We further hypothesized that the most prevalent AB in the *Microcystis* microbiome should co-occur with *Microcystis* in lake metagenomes. In contrast, they should not co-occur with another cyanobacterium frequently observed in Lake Champlain, *Dolichospermum*, which serves as a negative control. We first estimated normalized read counts and coverage of *Microcystis* and *Dolichospermum* in the 72 metagenomes from the Lake Champlain time series (Supplementary Fig. 9). We then estimated the Spearman correlations between *Microcystis* or *Dolichospermum* and each AB species or genus. The two cyanobacteria were weakly correlated with each other across the environmental metagenomes (Spearman ’ s *ρ* = 0.29 and *Q value* = 0.027). As expected, the nine most prevalent AB genera in the *Microcystis* microbiome were strongly correlated with *Microcystis* (Spearman ’ s *ρ* > 0.7, *Q value* < 0.001), and only weakly with *Dolichospermum* (Spearman ’ s *ρ* < 0.4, *Q value* > 0.001) with the exception of *Phenylobacterium* (Spearman ’ s *ρ* = 0.47, *Q value* < 0.001) which is nevertheless more strongly associated with *Microcystis* (Supplementary Figs. 10 and 11). The positive correlation between the most prevalent AB genera and *Microcystis* was also supported using an alternative correlation method, SparCC, which corrects for compositional effects in the relative abundance data (SparCC *r* > 0.4, *Q value* < 0.05) (Supplementary Table 10 and Supplementary Fig. 12). These significant positive correlations are consistent with close interaction between *Microcystis* and the most prevalent genera in their microbiome. Genera found at lower prevalence in *Microcystis* colonies (*e.g., Phycisphaerales bacterium* (unclassified genus) and *Telmatospirillum*) were poorly correlated with both *Microcystis* and *Dolichospermum*, suggesting weaker or transient associations (Supplementary Table 10 and Supplementary Fig. 10a).

Another AB belonging to the genus *Burkholderia* was quite prevalent in colonies but poorly correlated with *Microcystis* in metagenomes (present in the 40.37% of the colonies; Spearman ’ s *ρ* = -0.16, *Q value* = 0.343) suggesting contamination of colonies rather than a true ecological association. The genomes within this *Burkholderia* species showed nucleotide identity greater than 99% and short phylogenetic distances (0.0001), and was found in both Canadian and Brazilian colonies, suggesting a clonal lab contaminant rather than a batch or sampling effect. However, this was the only signal of contamination, suggesting that most of the other data reflect true associations.

Having already provided general support for phylosymbiosis, we sought to illustrate examples of associations between specific *Microcystis* genotypes and specific AB species. For example, *Rhodocyclaceae bacterium* G2 A13 was better correlated with genotype G05 than other *Microcystis* genotypes (Supplementary Fig. 13), consistent with the prevalence of this AB in 13 out of 14 colonies of genotype G05. In contrast, genotype G10 was poorly correlated with certain species within the genera *Roseomonas* and *Methylobacterium* (Spearman ’ s *ρ* < 0.38, *Q value* > 0.001). This illustrates how certain *Microcystis* genotypes have strong preferences for certain AB, while being unselective for others.

### Signatures of co-speciation between *Microcystis* and members of its microbiome

**Fig. 4:**
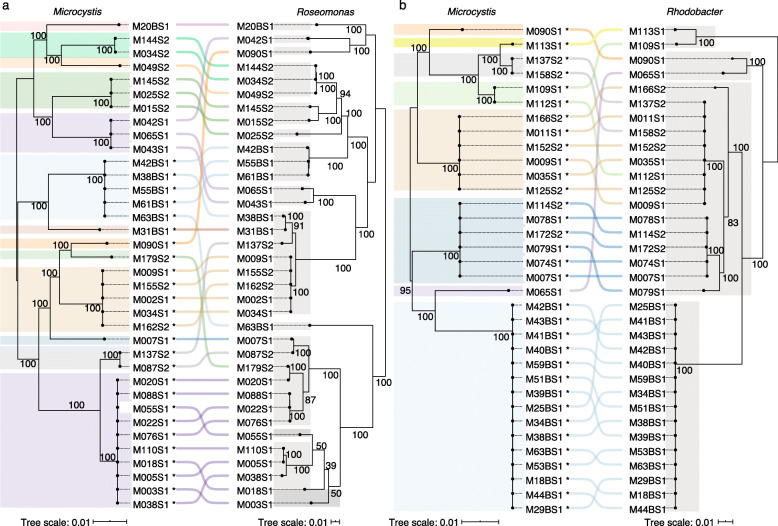
Co-phylogeny between *Microcystis* and two prevalent associated bacteria. **a** *Roseomonas* and **b** *Rhodobacter* core genome phylogenies were compared to the *Microcystis* core phylogeny. The lines between the two phylogenies connect genomes coming from the same *Microcystis* colony. The phylogenetic trees for *Microcystis*, *Roseomonas* and *Rhodobacter* were based on 706, 135 and 470 core genes, respectively. The different *Microcystis* genotypes are highlighted in colour, and the *Roseomonas* or *Rhodobacter* species in gray. The asterisks indicate the presence of the *mcy* cluster. While in a perfect co-phylogeny, the tree topologies of host and symbiont are identical; in these two comparisons, the co-phylogenetic similarity is imperfect but greater than expected by chance (ParaFit Global test, *P value* < 0.01)

Phylosymbiosis can arise due to vertical inheritance of microbiomes, or horizontal acquisition of microbiomes at each generation, provided that host lineages preferentially “match” with distinct microbiomes [24]. To assess the evidence for vertical inheritance of *Microcystis* AB, we used ParaFit to test for similarity between the *Microcystis* phylogeny and the phylogenies of the nine most prevalent AB genera strongly correlated with *Microcystis* but not with *Dolichospermum* in Lake Champlain (Supplementary Fig. 11). Each of these genera was represented by at least 12 high-quality draft genomes and was found in at least five different *Microcystis* genotypes. Significant co-phylogenetic signal suggests co-speciation of hosts and symbionts, consistent with a relatively long evolutionary history of association (*e.g.,* vertical descent). The ideal signal of co-phylogeny would be exactly congruent tree topologies for *Microcystis* and AB. In reality, we found tree topologies that were significantly similar (according to the ParaFit test), despite being non-identical. For example, *Roseomonas,* the second most prevalent AB genus in colonies, and *Rhodobacter*, the third most prevalent, had significant signatures of co-phylogeny (Fig. 4), while *Phenylobacterium* and *Chitinophagaceae* were borderline cases (Table 1). Overall, there was no clear tendency for stronger co-phylogeny with more prevalent AB, or with AB most correlated with *Microcystis* over time in Lake Champlain metagenomes (Table 1). However, such tendencies would be hard to discern in this relatively small sample size. As expected, the suspected contaminant *Burkholderia* A55 (*Burkholderia cepacia*) present in 40.37% of colonies, was poorly correlated with the presence of *Microcystis* in environmental metagenomes (*r* = − 0.16, *Q value* = 0.343), with no signal of co-phylogeny (*P value* = 0.732). Although co-phylogenetic signal was detectable in at least two of the most prevalent AB, the phylogenies are not identical (Fig. 4), suggesting a mixture of vertical and horizontal transmission. Even if horizontal transmission of AB among *Microcystis* lineages is likely, some degree of non-random host–microbiome matching must be occurring to explain the co-phylogenetic signal.

**Table 1:** Co-phylogeny analysis between *Microcystis* and the nine most prevalent associated bacterial genera within the *Microcystis* microbiome

Associated bacteria (AB) genus	Number of species per genus	Number of AB genomes used in the phylogeny	Prevalence of AB in colonies from Canada and Brazil	Correlation with *Microcystis* in Canada metagenomes (*r*^2^)	ParaFit test (*P values*)
*Phenylobacterium*	5	60	73.40%	0.759 *	0.072 (0.008)
*Roseomonas*	13	36	70.64%	0.835 *	0.009** (0.001)
*Rhodobacter*	4	34	46.79%	0.779 *	0.0018** (0.0002)
*Methylobacterium*	3	29	44.04%	0.809 *	0.729 (0.081)
*Pseudanabaena*	2	20	43.12%	0.766 *	0.153 (0.017)
*Rhodocyclaceae bacterium* G1	2	19	39.45%	0.769 *	0.225 (0.025)
*Rhodocyclaceae bacterium* G2	2	21	31.19%	0.776 *	5.355 (0.595)
*Chitinophagaceae bacterium*	3	22	26.60%	0.795 *	0.081 (0.009)
*Cytophagales bacterium*	3	16	22.94%	0.740 *	0.702 (0.078)

* Significant correlation coefficients (*Q* < 0.01)

** Significant *P values* (*P* < 0.01) (Bonferroni correction). Uncorrected *P values* are shown between parentheses

### Horizontal gene transfer (HGT) between *Microcystis* and its associated bacteria

**Fig. 5:**
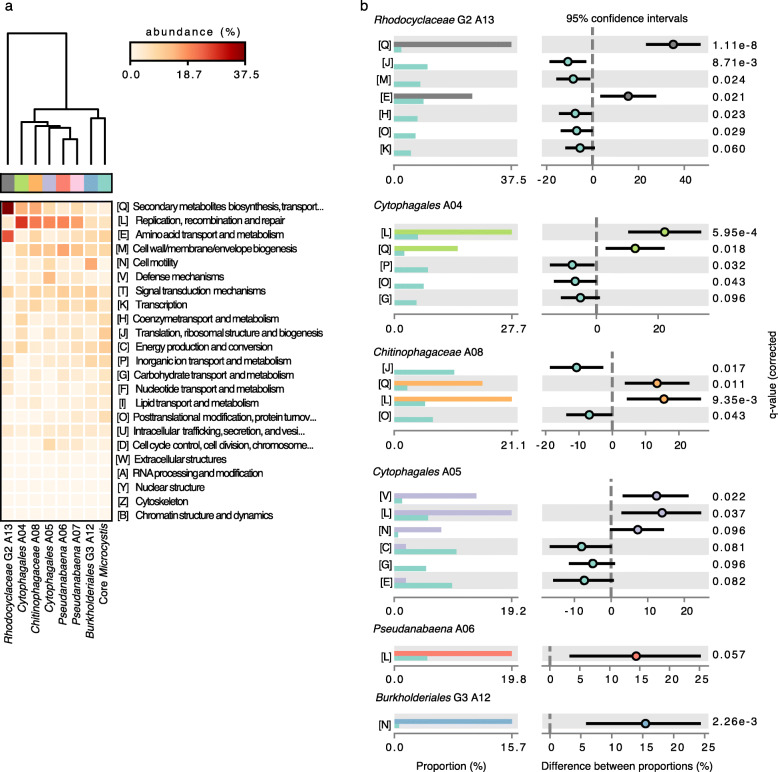
Inferred recent HGT between *Microcystis* and associated bacteria. Horizontally transferred genes between *Microcystis* and each AB species were inferred with a simple heuristic and annotated in 23 Clusters of Orthologous Groups (COGs) functional categories using EggNOG mapper (Methods). **a** Clustering analysis based on the relative abundance of the genes for each functional category, compared with the genes in the *Microcystis* core genome. **b** COG functions showing differential abundance between *Microcystis* core genes (turquoise) and the set of putative HGTs (other colors)

Unrelated bacteria sharing a common environment, such as the human gut, are known to engage in frequent horizontal gene transfer [40]. We hypothesized that *Microcystis* would also exchange genes with members of its microbiome, which share a similar physical niche—the colony milieu—for at least some period of time. We began by using a simple heuristic to look for similar gene sequences (≥ 99% amino acid identity) occurring in the *Microcystis* genome and at least one AB genome, as a proxy for relatively recent HGT events. Genome assembly and binning could affect this analysis by misplacing identical sequences either in *Microcystis* or in an AB genome, but not in both. To reduce this possible bias, we only considered a gene to be involved in HGT if it was present in at least four genomes (which would be unlikely to occur due to binning errors alone). We identified a total of 1909 genes involved in HGT between *Microcystis* and one of seven AB species: *Pseudanabaena* A06, *Pseudanabaena* A07*, Burkholderiales bacterium* G3 A12, *Rhodocyclaceae bacterium* G2 A13, *Chitinophagaceae bacterium* A08, *Cytophagales bacterium* A04 and *Cytophagales bacterium* A05. Compared to the *Microcystis* core genes, these candidate HGTs are enriched in functions related to secondary metabolite biosynthesis, replication and recombination, and defense mechanisms (Fig. 5). As a control, we repeated the HGT inference using the likely contaminant *Burkholderia* A55 genome instead of *Microcystis*. We identified 558 putative HGT events, of which 523 involved species were not found to engage in HGT with *Microcystis: Methylobacterium* A30, *Rhodocyclaceae bacterium* G1 A54 and *Cupriavidus* A44. This suggests that *Microcystis* engages in more HGT with its microbiome than a random expectation (*i.e.,* with a contaminant genome), and allows us to conservatively estimate the false-positive rate of HGT detection at 523/(523 + 1909), or 22%. Despite the significant noise, we expect the broad gene functional categories and specific AB involved in HGT with *Microcystis* to be relatively robust.

As an additional validation of our HGT heuristic, we used Metachip, which uses phylogenetic incongruence in addition to a sequence identity threshold [41]. Metachip identified the same seven AB genera involved in HGT with *Microcystis* based on our simple heuristic, except for *Rhodocyclaceae bacterium* G2. Metachip is much more conservative, identifying only 46 gene families involved in HGT (Supplementary Table 11). Of these gene families, 31 were also identified by our heuristic method, suggesting they are high-quality candidates.

### Cellular functions encoded by members of the *Microcystis* microbiome

In contrast to genes shared by HGT, there may be a genetic division of labour between *Microcystis* and its microbiome, which would then be expected to encode different and complementary sets of gene functions. To compare these gene functions, we first characterized orthologous genes using the Kyoto Encyclopedia of Genes and Genomes (KEGG) orthologues (KO) in both *Microcystis* and its microbiome. We then used ANTISMASH [42] to identify gene clusters involved in the biosynthesis of cyanopeptides and other pathways of interest. We further validated the presence of biosynthetic pathways like biotin, cobalamin, nitrogen fixation and carotenoids with gapseq [43]. As expected for distantly related bacteria, *Microcystis* genotypes and AB encode distinct sets of gene functions based on the presence/absence of annotated genes (Supplementary Fig. 14). Bacteria from the same Phylum tend to cluster together in terms of their functional gene content. For example, *Microcystis* clusters with its fellow cyanobacteria *Pseudanabaena,* while *Bacteroidetes* (*i.e., Cytophagales bacterium* and *Chitinophagaceae bacterium*) formed a distinct cluster (Supplementary Fig. 14).

*Roseomonas* and *Rhodobacter*, which show co-phylogeny with *Microcystis* but appear not to engage in significant amounts of HGT, are prime candidates for functional complementarity to have evolved and be maintained with high partner fidelity. Both these genera contain carotenoid biosynthesis pathways different from those found in *Microcystis* genomes (Supplementary Table 12). For example, neurosporene, spirilloxanthin and spheroidene pathways are present in *Rhodobacter* but not in *Microcystis.* Carotenoid pigments like zeaxanthin are generally produced by *Microcystis* for their photoprotective properties and their capacity to improve the efficiency of photosynthesis [44]. Indeed, in our *Microcystis* genomes, we found the complete pathways for the biosynthesis of zeaxanthin, echinenone and intermediaries in the biosynthesis of carotenoids like trans-lycopene. It is tempting to speculate that the *Microcystis* microbiome could be involved in the production of additional carotenoids that could provide additional photoprotection under certain environmental conditions; this deserves further study. Certain AB (*i.e., Roseomona*s, *Rhodobacter* and *Methylobacterium*) also encoded genes involved in anoxygenic photosynthesis (absent in *Microcystis*) and the transport of rhamnose, D-xylose, fructose, glycerol and a-glucoside (Supplementary Table 13), which could also complement the metabolic repertoire of *Microcystis* [16].

To test for potential metabolic interactions between *Microcystis* and its microbiome, we reconstructed genome-scale metabolic models with CarveMe [45] and used SMETANA [46] to identify exchanged metabolites that are essential for the survival of the community (Methods). We performed these analyses on *Microcystis* genotype G01 and its microbiome of five AB, which are among the most prevalent across the genus. This analysis suggests that *Microcystis* is a major recipient (but not a donor) of metabolites from AB, including amino acids and nutrients (Supplementary table 14A). As a negative control, we analyzed the interactions between *Microcystis* and taxa that did not co-occur (based on SparCC and Spearman correlation results), yielding no metabolic exchanges inferred by SMETANA. *Microcystis* is unable to fix nitrogen, but it contains pathways to assimilate nitrate and ammonia. Previous studies have suggested that *Microcystis* might rely on its microbiome for nitrogen [16, 47]. Although we found no complete pathways for nitrogen fixation in AB genomes, metabolic reconstruction suggests that *Methylobacterium, Roseomonas* and *Rhodobacter* might provide *Microcystis* with nitrate, nitrite and ammonium (Supplementary Table 14A). In a second SMETANA “global” analysis of species pairs, we found that *Roseomonas* is the AB with the lowest metabolic resource overlap (MRO score) and the highest metabolic interaction potential (MIP score), suggesting metabolic complementarity (Supplementary Table 14B). *Roseomonas* was the only AB with a lower MRO score and a higher MIP score than observed in negative controls (Supplementary Table 14C). Together, these results suggest the potential for significant metabolic exchange between *Microcystis* and its microbiome, particularly *Roseomonas.*

## Discussion

By combining single colony sequencing and metagenome analysis, we explored the genetic diversity of both *Microcystis* and its microbiome, and their variation over time in Lake Champlain, Canada and the Pampulha reservoir in Brazil. We revealed a higher diversity of *Microcystis* genotypes than previously described [48], and a strong signal of phylosymbiosis between the host and its microbiome. Despite the absence of a strict core microbiome, the overall microbiome community composition tends to change proportionally along the *Microcystis* host phylogeny—a hallmark of phylosymbiosis [24]. Although some members of the microbiome might be loosely associated, some—notably *Roseomonas* and *Rhodobacte*r—appear to be relatively stable over evolutionary time (taking phylogenetic branch length as a rough proxy for time). These two genera have been previously reported to be correlated with *Microcystis* in environmental samples [49, 50]. Whether these associations are beneficial to one or both partners remain to be seen.

There has been some debate about whether *Microcystis* colonies form by clonal cell division, or by aggregation of (potentially distantly related) cyanobacterial cells [21, 51]. Our results suggest that clonal cell division is more likely, based on our observation of much greater genetic variation in the *Microcystis* genome between than within colonies of the same genotype. This finding applies to the relatively large colonies we sampled from eutrophic lakes, and results could differ for smaller colonies, under oligotrophic conditions [32], or under strong grazing predation, which could affect colony structures [52]. Because DNA extraction is more likely to have failed for smaller colonies, our collection likely underrepresented smaller colonies, which could represent distinct genotypes. However, 93.5% of *Microcystis* metagenomic reads from Lake Champlain were recruited to our collection of colony genomes at 99% nucleotide sequence identity, suggesting that the majority of natural *Microcystis* diversity is represented in our sample of colonies. Of course, these results are specific to Lake Champlain and Pampulha, and should be replicated in other lakes under different environmental conditions (*e.g.,* oligotrophic lakes).

Phylosymbiosis and co-speciation appear to be relatively common and strong in mammalian gut microbiomes [24, 25], and even in the more environmentally exposed coral microbiome [24, 25]. It is unclear if such tight and evolutionarily stable associations would apply to *Microcystis* and its AB, or if more transient interactions would prevail. While the idea of a *Microcystis* microbiome has been suggested previously based on bulk metagenomic and amplicon sequencing from lakes [16, 53], here we refine the *Microcystis* microbiome concept beyond co-occurrence patterns to physical association within a colony. We found that the most prevalent AB from individual *Microcystis* colonies also tend to co-occur with *Microcystis* over time in Lake Champlain. The composition of the microbiome varies along the *Microcystis* phylogenetic tree, consistent with phylosymbiosis and relatively long-term evolutionary associations. At least two AB show a significant co-phylogenetic signal, suggesting co-speciation with *Microcystis.* Therefore, although possibly not as strong as in mammals or even coral, co-phylogeny is a feature of at least certain members of the *Microcystis* microbiome, yet co-phylogeny is unlikely to explain most of the signal of phylosymbiosis. Alternatively, phylosymbiosis can arise as a consequence of shared biogeography between hosts and microbiomes [54], and we do observe distinct microbiomes in Brazil and Canada. However, we found evidence for phylosymbiosis within a single lake in Canada, suggesting that factors other than biogeography—such as host–microbiome trait matching—are likely at play.

As expected for distantly related bacteria, *Microcystis* and its AB encode different functional gene repertoires, some of which could be complementary and mutually beneficial. We found that both *Microcystis* and AB have biosynthetic functions for a wide range of carotenoids. Metabolic pathways for carotenoids in *Microcystis* differed from those found in AB. Carotenoids act as antioxidants and may broaden the photosynthetic light absorption spectrum [55, 56]. In a previous study, heterotrophic bacteria producing carotenoid pigments showed higher survival to environmental stressors (*e.g*., solar radiation) than their unpigmented counterparts [57]. Further studies are required to understand whether the additional carotenoid pathways in the AB enhance the host photo protection.

Horizontal gene transfer (HGT) is relatively common in bacteria, and may occur among unrelated bacteria [58] particularly when they share an ecological niche such as the human gut [40]. *Microcystis* is physically associated with its microbiome for at least part of the colony life cycle, and we hypothesized that HGT could occur within colonies. Using two methods to detect HGT, we found evidence for gene transfers between *Microcystis* and at least six different species of AB: two species of *Pseudanabaena,* two *Cytophagales*, one *Burkholderiales*, and one *Chitinophagaceae* species. Certain gene functions, including secondary metabolite biosynthesis, replication and recombination, and defense mechanisms, are particularly prone to HGT between *Microcystis* and its microbiome, and the ecological functions of these genes deserves further study. Notably, prevalent AB with evidence of co-phylogeny with *Microcystis* (*Roseomonas* and *Rhodobacter*) shared relatively few (less than seven) HGT events with *Microcystis*. This counter-intuitive result could be explained if these co-phylogenetic associations are relatively ancient, but our HGT detection is biased toward recent events. Alternatively, it is possible that HGT is more likely among less intimately associated AB, whereas more intimate associations would select for only one, but not both partners, to encode the gene. This would also require that metabolites are shared between partners. To explain this result, we hypothesize that such long-term associations might favour the loss of redundant genes, as predicted by the Black Queen Hypothesis [59]. In other words, a gene needs to be encoded by only one partner, provided that gene products or metabolites are shared between partners. Such metabolic interdependencies appear to be relatively strong between *Microcystis* and *Roseomonas.* Therefore, even if HGT does occur between partners, we would not expect to find the same gene redundantly encoded in both partners. These evolved co-dependencies would further reinforce partner fidelity and could help explain the co-phylogenetic signal between them.

## Conclusions

Overall, our results provide evidence for eco-evolutionary associations between *Microcystis* and its microbiome. Some members of the microbiome may be more tightly associated than others, and based on their gene content, we hypothesize that they might provide complementary functions to *Microcystis.* Such complementary functions can be encoded by a variety of species, such that there is no strict species- or genus-level core microbiome. These hypotheses could be tested in experimental co-cultures, which have recently shown how the *Microcystis* microbiome can alter its competitive fitness against eukaryotic algae [60]. Such experiments could be extended to the combinations of *Microcystis* genotypes and AB which we have shown to be intimately associated in nature.

## Materials and methods

### Sample collection and DNA extraction for colonies and metagenomes

To access the genomic diversity of *Microcystis* in Lake Champlain and Pampulha reservoir, 346 individual *Microcystis* colonies were isolated across the bloom season in 2017 and 2018 (July to October in Quebec, Canada (45° 02’ 44.86” N, 73° 07’ 57.60” W) and April to November in Minas Gerais, Brazil (19° 55’ 09” S and 43° 56’ 47” W)) during 2018. Colonies were isolated from surface water samples (~ 50 cm depth) after concentration using a plankton net (mesh size 20 μm). One litre of concentrated water was collected and stored at 4 °C for a maximum of 36 h until colony isolation. Colonies were isolated using micropipes, sterile medium (Z8 medium) and a microscope (Nikon E200 Eclipse). Each colony was washed 15–20 times using sterile Z8 medium and stored at − 80 °C until DNA extraction. The DNA extraction was performed directly on each colony using the ChargeSwitch® gDNA Mini Bacteria Kit. Two additional steps were added to ensure the rupture of the *Microcystis* colonies and cells (see Supplementary Methods). Briefly, each colony was added to a tube containing 50 mg of beads (PowerBead tubes, glass 0.1 mm, Mo-bio), incubated with lysis solutions, and then vortexed using the TissueLyser LT (Qiagen) for 3 min at 45 oscillations per second. The tube was then centrifuged for 1 min at 9000 rcf. This procedure yielded DNA for 109 colonies (Supplementary Figs. 15 and 16), sequenced as described below. Matched water samples were collected at the same place and time as colonies, spanning 16 time points (Supplementary Table 15). Water temperature and pH were also measured at each sampling point.

For metagenomic sequencing, a total of 72 lake water samples were collected over 10 years (2006 to 2018) during the ice-free season (April to November) from the photic zone of Missisquoi Bay at two different sites (littoral and pelagic) of Lake Champlain, Quebec, Canada (45° 02’ 45” N, 73° 07’ 58” W). Lake water was filtered, and DNA was extracted using a Zymo Kit (Zymo, D4023) as described previously [61]. The filtration was performed the same day of the sampling, using between 50 and 250 mL of water samples, depending on the amount of biomass, onto 0.2 μm hydrophilic polyethersulfone membranes (Millipore, Etobicoke, Ontario, Canada). Samples were obtained at relatively low frequency between 2006 and 2016, and at higher frequency (approximately weekly or more often) during bloom periods between 2015 and 2016 (Supplementary Table 3). Note that no colonies were isolated from 2006 to 2016. Water samples corresponding to six sampling points from Minas Gerais Brazil were also collected for DNA extraction and metagenome sequencing. Environmental variables were measured for each sample. Sample water were collected (50 mL) for measuring nutrients (DN, DP, TP and TN), except for the samples from Brazil (Supplementary Table 3) [61].

### DNA sequencing of single colonies and metagenomes

DNA extracted from *Microcystis* single colonies was sequenced using the Illumina HiSeq 4000 platform with 150 bp paired-end reads. The sequencing libraries (with average fragment size 360 bp) were prepared using the NEB (New England Biolabs®) low input protocol. The DNA extracted from filtered bulk lake water for each sampling point (2017 and 2018) from Canada and Brazil were sequenced using Illumina NovaSeq 6000 S4 platform with 150 bp paired-end reads. The earlier lake water samples from a previous long-term experiment in Lake Champlain (2006 to 2016) were sequenced using Illumina Hiseq2500 with 125 paired-end reads (Supplementary Table 3). The quality score and number of reads per sequenced sample are reported in Supplementary Table 16. Quality scores were calculated using the program fastp (v0.20.1) [62].

### Metagenome assembly and genome binning

For each individual *Microcystis* colony, the sequencing reads were filtered and trimmed using Trimmomatic (v0.36) [63] then assembled with MEGA-HIT (v1.1.1) [64], producing contigs belonging to both *Microcystis* and AB (see Supplementary Fig. 17 showing the distribution size of the contigs). We performed manual binning on each individual colony assembly separately using Anvi’o (v3.5) with default parameters, only including contigs larger than 2500 bp, as described previously [31, 65]. This contig size cutoff of 2500 bp improves binning based on k-mer frequencies, and is standard for metagenomic analyses of microbial communities from diverse environments [66–68]. Briefly, for each sample, we generated a contig database, and the reads were mapped back to contigs using bowtie2 (v2.3.4.3) [69]. The contigs were interactively manually curated in Anvi’o based on the read coverage from the mapping, tetranucleotide frequency, GC content and taxonomic annotations obtained from centrifuge (v1.0.3) [70], and using the program ‘anvi-profile’ as implemented in Anvi’o [65]. The quality of each resulting MAG was estimated using Anvi’o and based on a collection of 139 bacterial single-copy core genes [33, 65]. From the 109 colonies, 500 MAGs were identified (defined as having completeness ≥ 70% and contamination ≤ 10% as in Lee *et al*. [71]) (Supplementary Tables 1 and 5). We used the program dRep (v3.2.0) with default parameters to investigate the presence of redundant MAGs in each colony metagenomes [72]. However, within an individual colony, we found no redundant MAGs. MAGs were annotated using Prokka (v1.14.0) [73]. Pairwise average nucleotide identity (ANI) values between genomes were estimated using pyani (v0.2.8) [74, 75]. MAGs were classified into species-level taxonomic groups at a threshold of ANI ≥ 96% (Supplementary Tables 5 and 17). The MAGs were assigned species and genus names using BLASTp of the recA and RpoB proteins against the NCBI database, and refined using the Genome Taxonomy Database Toolkit (GTDB-Tk) (v1.0.2), which uses a set 120 universal bacterial gene markers [76]. To assess the presence of eukaryotic microbes, the contigs in the uncomplete or MAGs with completeness < 70% and contamination > 10% were classified taxonomically using the program CAT (v5.2.3) (Supplementary Table 18) [77].

For each taxonomic group, we selected at least two representative sequence types (for a total of 138 genomes), from which we inferred a maximum likelihood phylogenetic tree based on the core gene alignment using RAxML (v8.2.11) [78]. The core genome was estimated using panX (v1.5.1). Core genes were defined as those genes present in at least the 80% of sampled genomes (e value < 0.005) [79]. Each of the resulting 62 core genes was alignment using muscle (v3.8.3) [80]. Filter.seqs from mothur (v1.41.3) was used to remove the gaps per each gene alignment [81]. Individual alignments were concatenated into a single alignment (16,400-bp long) input into RAxML. For the phylogenetic reconstruction, we used a GTR-GAMMA substitution model with 100 bootstraps, and best maximum likelihood tree inference.

### Assessment of the *Microcystis* genotype diversity in freshwater colonies

A core genome was also estimated for the 109 *Microcystis* genomes and 122 NCBI references genomes (Supplementary Table 1 and 19). The resulting alignment of the 115 core genes was degapped (68,145-bp long) and used to infer an ML phylogeny using RAxML as we described previously. Two outgroups (*Anabaena variabilis* ATCC29413 and *Synechocystis* sp. PCC6803) were included. Based on ANI values greater or equal to 99%, the monophyletic clades of *Microcystis* genomes were classified into 18 genotypes (Supplementary Table 2). A phylogenic tree without the outgroups is also included in Supplementary Fig. 18. Monophyletic clades and tree branches remained similar.

### Assessment of the *Microcystis* genomic (within-colonies) variation versus intra-genotype variation (between colonies)

We first confirmed that *Microcystis* is haploid, as polyploidy has been observed among other cyanobacteria [82]. We estimated ploidy variation in *Microcystis* colonies using k-mer frequencies and raw sequences. We first mapped the reads of each colony (containing reads from both *Microcystis* and its microbiome) to a *Microcystis* reference genome using BBmap with minimum nucleotide identity of 99% [83]. Mapped reads were extracted using Picard (*http://broadinstitute.github.io/picard/*) and analyzed using Genomescope and Smudgeplot (*https://github.com/tbenavi1/genomescope2.0**;* *https://github.com/KamilSJaron/ smudgeplot*). All colonies appeared to be haploid, with a low rate of heterozygosity that could be due to paralogs.

To determine whether *Microcystis* colonies were likely formed by clonal cell division or cell aggregation, we called SNVs within colonies and between colonies of the same genotype. As a point of comparison, we also called SNVs that occurred over a period of approximately 6 years in nineteen laboratory cultures of *Microcystis* with genome sequences reported previously [31]. We used snippy (v4.4.0) (*https://github.com/tseemann/snippy*) with default parameters to call SNVs. Genotypes represented by only one sampled colony were excluded from the analysis (G02, G04, G09, G11, G12, G16 and G18).

SNV calling within and between colonies was executed by mapping reads against reference genomes. This was done independently for each genotype. We selected at least four reference genomes per genotype when possible. SNVs within colonies were detected by mapping the reads of the references to their respective genome assemblies. SNVs between colonies were detected by mapping the reads of different colonies of the same genotype to the genome assemblies of the references. We ignored positions where the reference nucleotide was poorly supported (threshold percentage for the minor variant < 14.4%; mean = 1.1%) by the reads in both the within- and between-colony read mapping analyses because these were considered to be assembly errors.

### Identifying associated bacterial genomes in colonies

Non-*Microcystis* MAGs from each colony were classified in 72 species based on taxonomical analysis and ANI values ≥ 96%. Because individual assemblies could affect MAG completeness, we created a custom database of the 59 AB genomes from Quebec, and another database for the 18 species from Brazil. Using MIDAS (v1.3.0) [39], we mapped the reads from each colony (downsampled to 8,000,000 reads per colony) against the custom databases to estimate the relative abundance and coverage for each of the 72 AB species. These custom databases contain one representative genome per species, chosen as the highest quality MAG within that species. We defined a species to be present when it had a genome-wide average depth of coverage of 1× or more. This allowed us to generate a matrix of AB presence or absence across colonies. To take into account both assembled and unassembled taxa, the microbiome composition was assessed using two additional approaches: (1) by mapping the reads against the complete MIDAS reference genome database containing 5952 species and (2) by using the read counts and taxonomical annotation based on the program Kaiju (v1.8) with default parameters [84].

### *Microcystis’* microbiome composition variation according to environmental variables and host genotype

The following statistical analyses were conducted in R (v3.6.2). We first performed a distance-based RDA using the *capscale* function from the R *vegan* package (v2.5.61, [85]) with the square root of the Bray–Curtis distance from a coverage table describing the composition of the *Microcystis* microbiome for each genotype. The variables included genotype information, presence/absence of *mcy* genes, temperature, pH, site (Canada or Brazil) and the temporal variables years and months. In a second approach, we calculated the beta diversity using the same dissimilarity distance and tested the differences in *Microcystis* microbiome structure variation using permutational multivariate analysis of variance (PERMANOVA, [86]) with the adonis() function from the R *vegan* package. As PERMANOVA tests can be sensitive to dispersion, we have also tested for dispersion in the data by performing an analysis of multivariate homogeneity (PERMDISP, [87]) with the permuted betadisper() function of the *vegan* package).

We quantified phylosymbiosis by comparing the phylogenetic distance matrix of *Microcystis* genotypes and the microbiome composition distance matrix using a Mantel test (999 permutations, Spearman correlation) and the protest() R function to test the non-randomness between these two matrices (999 permutations) (R *vegan* package). The pairwise phylogenetic distance matrix was estimated using the RAxML tree of the *Microcystis* core genome and the cophenetic.phylo function of the ape R package (v5.3) [88]. The results based on the extended MIDAS reference database and Kaiju taxonomic assignments are described in the supplementary material (Supplementary Table 8).

### *Microcystis* genotypic diversity from metagenomic samples

*Microcystis* genomes from Quebec and Brazil were classified into 14 and four genotypes, respectively. This genotype classification was based on pairwise genome similarities greater or equal to 99%. Using the *Microcystis* genotypes and the software MIDAS (v1.3.0) [39], we built two custom gene marker databases for the *Microcystis* genotypes (15 universal single-copy gene families), one for genotypes from Quebec and the other for genotypes from Brazil.

Using MIDAS and the custom databases, we estimated the relative abundances, the read counts and the read coverage of the *Microcystis* genotypes in 72 shotgun metagenomes from Lake Champlain, Quebec (62 metagenomes from a long-term experiment (2006 to 2016, excluding 2007 and 2014), plus 10 metagenomes from 2017 and 2018). Due the low number of *Microcystis* genotypes and metagenomes (6 sampling points for Brazil during 2018) from Brazil, these samples were not formally analyzed. Metagenomic reads with similarity greater or equal to 99% were mapped against the MIDAS database of *Microcystis* genotypes. We used 14,000,000 reads per metagenome after downsampling to the lowest-coverage metagenome (Supplementary Table 3). The metagenome sequencing from Brazil were mapped against a separate MIDAS database of the four *Microcystis* genotypes from Brazil (Supplementary Fig. 19).

To test if the 14 *Microcystis* genotypes identified in the colony genomes are representative of the diversity present in the Lake Champlain metagenomes, we first mapped the downsampled metagenomic reads to a custom database including a single reference *Microcystis* genome (M083S1) (alignment identity cutoff = 96%), and also mapped the reads to the database including all the 14 genotypes (alignment identity cutoff = 99%). By using a cutoff value equal to 96%, we expect to recover most sequences from the *Microcystis* genus, regardless of which genotype the reads come from. We recovered 102,608 reads at 99% identity and 109,729 at 96%, showing that the 14 genotypes (defined at 99% identity) account for 93.5% of the *Microcystis* reads in the metagenome samples. Additionally, we observed that the total coverage using all the *Microcystis* genotypes (alignment identity cutoff = 99%) and the total coverage using a single *Microcystis* genome as a reference (alignment identity cutoff = 96%) are nearly perfectly correlated (correlation coefficient *r* = 1, *P* < 2.2e–16) (Spearman correlation) (Supplementary Fig. 20).

### *Microcystis* genotypic diversity variation according to environmental variables

To determine the factors that explain the variation in *Microcystis* community composition, we used a dataset of 42 metagenomes and 14 genotypes from Lake Champlain. Metagenomes with incomplete metadata were excluded. We focused on Lake Champlain as we observed a greater diversity of *Microcystis* genotypes compared to Brazil, including both microcystin-producing and non-producing genotypes. Statistical analyses were performed in R (v3.6.2). We first used dbRDA with the square root of the Bray–Curtis distance matrix to investigate *Microcystis*–environment relationships [89, 90] (capscale function from vegan R package, R *vegan* package). Variables were pre-selected using the ordiR2step R function (R *vegan* package) (see Supplementary Methods). The environmental matrix variables included: total phosphorus in micrograms per litre (μg/L) (TP), total nitrogen in μg/L (TN), soluble reactive phosphorus in μg/L (DP), dissolved nitrogen in μg/L (DN), 1-week-cumulative precipitation in millimetres (mm), 1-week-average air temperature in Celsius, temporal variables (Years, Months and Season) and sampling sites within Lake Champlain (Pelagic or Littoral) (Supplementary Table 3) [61]. To determine the significance of constraints, we used the anova.cca() function from the R *vegan* package.

We also calculated the beta diversity between groups of samples using the Phyloseq R package (v1.30.0) [91] and the square root of Bray–Curtis distance. We used nonmetric multi-dimensional scaling (NMDS, from the phyloseq package that incorporates the metaMDS() function from the R vegan [85, 92, 93] package to ordinate the data. Differences in community structure between groups were tested using permutational multivariate analysis of variance (PERMANOVA, [86]) with the adonis() function. As described previously for similar analysis, we also tested for differences in dispersion between groups (genotypes) with the permuted betadisper() function.

### Correlations between and *Microcystis* and its microbiome in freshwater metagenomes from Canada

Using the 59 species identified in the *Microcystis* microbiome from Canada and the software MIDAS, we built a custom gene marker database of 15 universal single-copy gene families. This database also included a reference genome from *Microcystis* (M083S1) and two *Dolichospermum* reference genomes (*D. circinale* AWQC131C and AWQC310F). Using MIDAS, we estimated the relative abundances, reads count, and the read coverage of each AB species in 72 shotgun metagenomes from Quebec, Canada. Reads were mapped against the custom database including the AB species. A cuff-off value of nucleotide identity greater or equal to 96% was used for the read mapping. By merging the values (coverage and read counts) for species within the same genus, obtained coverage and read counts at the genus level, for 32 genera of AB. We used the Spearman correlation to investigate patterns of co-occurrence between *Microcystis*, *Dolichospermum* and the AB species and genera in environmental metagenomes. First, the read counts in the matrices containing the genera and species were used to estimate the Spearman correlation values (*r*) and *P* values between pair of species or genera by using the rcorr() function of the Hmisc (v4.3.0) R package [94]. We also calculated Spearman correlations on the coverage values, yielding similar results. *P* values were corrected to control the false discovery rate using the qvalue() function from the qvalue (v2.18.0) R package. We also estimated the correlation between *Microcystis* and the AB using the software FastSpar (v0.0.10) [95]. This method is a faster implementation of the Sparse Correlation for Compositional Data algorithm (SparCC) [96]. The significance of the test was evaluated using 100 permutations and a bootstrap of 1000. In general, the most prevalent AB taxa in *Microcystis* colonies had significant correlation (*P* < 0.05) with *Microcystis* using both Spearman and SparCC.

### Co-phylogeny between *Microcystis* and members of the microbiome

The nine most prevalent AB genera were selected for co-phylogeny analysis, which would be underpowered to detect phylogenetic associations with low-prevalence bacteria (*i.e.,* small phylogenies). Core genomes were generated using panX and core alignments were computed as described above, for each AB genus. Phylogenic core genome trees were built individually for each genus using RAxML under the GTR-GAMMA substitution model [78]. Support values of the tree nodes were estimated using 100 bootstrap replicates. Patristic distances (pairwise distances between pairs of tips on a tree) for the *Microcystis* and AB phylogenies were estimated using the cophenetic.phylo() function from the ape R-package [88]. The *Microcystis* core genome tree and the tree of the AB were compared using Parafit test (parafit() function of the ape R package) (see Supplementary Methods) [88, 97]. Co-phylogeny trees were built using the function cophylo() from the phytools R package [98].

### Recent HGT between *Microcystis* and associated bacteria

To infer recent HGT events between *Microcystis* and AB, we first inferred the pangenomes for each combination of one AB and *Microcystis*, and repeated this for the 72 AB species. Core and accessory genes with a minimum percentage identity for blastp equal to 99% were identified. We retained those clusters of genes present in at least four genomes, and present in both AB and *Microcystis*. The remaining putatively horizontal transferred genes were annotated in 23 COG (clusters of orthologous groups) categories using eggNOG-mapper (v2.0.1) [99]. Using the package STAMP (v2.1.3) and a chi-squared test, we estimated if there were statistical differences in the COG categories between *Microcystis* core genes and the putative horizontally transferred genes [100]. *P* values were corrected using the Benjamini–Hochberg (controlling the false discovery rate) method. We also estimated HGT events between *Microcystis* and associated species using a second method, Metachip (v1.8.2) (default parameters). The Metachip approach uses both the best match approach (blastn) and a phylogenetic approach to infer HGT (reconciliation between a gene tree and its species tree) [41].

### Gene functional annotation

The *Microcystis* and AB genomes were functionally annotated using enrichM (v0.5.0) (*https://github.com/geronimp/enrichM*) [101]. A PCA based on the presence/absence of KEGG Orthologous genes (KO) in *Microcystis* and AB genera was generated using the option ‘enrichment’ in enrichM. Genome groups (*Microcystis* vs each AB genus) were compared using the same option. KEGG modules differentially abundant in *Microcystis* or the AB genus were filtered based on a completeness greater or equal to 70%. Additionally, we used the program gapseq (v1.1) with default parameters and the MetaCyc database [43, 45] to validate the presence the metabolic pathways involved in the biosynthesis of biotin, cobalamin, thiamine, carotenoid and nitrogen fixation.

*Microcystis* and AB genomes (109 *Microcystis* and 391 associated genomes) were annotated using Roary (v3.13.0) [102]. The resulting genomes in GenBank format were used to predict the biosynthetic gene clusters (BGCs) using default parameters (--taxon bacteria --cb-general --cb-knownclusters --cb-subclusters --asf --pfam2go --smcog-trees --genefinding-tool prodigal-m) in antiSMASH (v5.1.2) [42, 103]. The BIG-SCAPE package (v1.0.1) with default parameters analyzed the ANTISMASH BGCs and based on a similarity network classified them into Gene Cluster Families (GCFs) [104]. BGCs were classified in BiG-SCAPE classes (*e.g.*, polyketide synthases nonribosomal peptide synthetases (NRPSs), post-translationally modified peptides (RiPPs) and terpenes. A total of 2395 BGCs were identified in 415 genomes.

### Metabolic pathway reconstruction

We reconstructed genome scale metabolic models from the genomic sequences of one *Microcystis* genotype (G01) and its microbiome composed of *Roseomonas*, *Rhodobacter*, *Methylobacterium*, *Phenylobacterium and Pseudanabaena*, which are among the most prevalent AB for *Microcystis* as a whole. We used MAG annotations from prokka (v1.14.0) [73] as input for CarveMe (v1.5.0) [105] to generate the models, with default parameters. We next assessed the metabolic interactions using SMETANA (v1.1.0) [46] excluding inorganic compounds which we assumed are generally present in the water system, and using the –molweight parameter to minimize the total mass of the compounds in the media, which is less prone to degenerate solutions. SMETANA is a flux balance analysis-based simulation tool that first estimates the minimal amount of nutrients required for the community to survive. A “detailed” analysis estimates the interspecies dependencies under a minimal constrained nutritional environment, by calculating a species coupling score (SCS), metabolite uptake score (MUS), metabolite production score (MTS) and a SMETANA score (a combination of the three other scores). A second “global” analysis characterizes the general interactions of all members in noninteracting and interacting communities by calculating the metabolic resource overlap (MRO) and metabolic interaction potential (MIP) [46]. As a control, we analyzed the interactions with taxa (*i.e*., *Phycisphaerales*, *Burkholderia* and *Acidobacteriaceae*) that were not co-occurrent with *Microcystis* in metagenomes based on SparCC and Spearman correlation results. The output was empty meaning that there were no cross-feeding interactions between *Microcystis* and these taxa. When at least one species depends on the others to survive, cross-feeding interactions are listed in the SMETANA detailed output file.

## Supplementary Information

**Additional file 1.** Supplementary methods. DNA extraction colonies.

**Additional file 2.** Supplementary methods. dbRDA phylosymbiosis (R script).

**Additional file 3.** Supplementary methods. Co-phylogeny (R script).

**Additional file 4.** Supplementary figures.

**Additional file 5: Table S1.***Microcystis* genome characteristics.

**Additional file 6: Table S2.** ANIm and ANIb values.

**Additional file 7: Table S3.** Metadata for metagenomic samples from Lake Champlain and Pampulha Reservoir.

**Additional file 8: Table S4.** SNVs within and between *Microcystis* colonies. SNVs accumulated in 19 *Microcystis* laboratory cultures after ~6 years of culture are also included.

**Additional file 9: Table S5.** AB genome characteristics.

**Additional file 10: Table S6.** Kaiju core species according to various read counts cut-off.

**Additional file 11: Table S7.** dbRDA and PERMANOVA results.

**Additional file 12: Table S8.** (A) PERMANOVA and (B) Phylosymbiosis results both based on the microbial community composition that includes assembled and non-assembled taxa.

**Additional file 13: Table S9.** Single-copy taxonomic marker genes for MIDAS analysis.

**Additional file 14: Table S10.** Spearman and SparCC correlations.

**Additional file 15: Table S11.** Gene families involved in HGT using Metachip. The table shows the genes identified with Metachip, and 31 genes families that were also identified with an additional method.

**Additional file 16: Table S12.** Presence/absence of certain metabolic pathways involved in the biosynthesis of biotin, cobalamin, thiamin and carotenoid, and nitrogen assimilation.

**Additional file 17: Table S13.** KEGG modules present in AB.

**Additional file 18: Table S14.** Metabolic exchange analysis. (A) SMETANA output from a modeled community of *Microcystis* genotype G01 (M007S1) and the five members of its microbiome in minimal media. (B) Pairwise SMETANA analysis of *Microcystis* and five members of its microbiome in complete media. (C)Negative control pairwise SMETANA analysis of *Microcystis* and three non-associated bacteria in complete media.

**Additional file 19: Table S15.** Colonies sequenced per each sampling time point.

**Additional file 20: Table S16.** Read quality scores for the *Microcystis* colonies and the environmental metagenomes. Quality scores for the colonies (A and B) and for the lake metagenomes (C and D).

**Additional file 21: Table S17.** ANIm between AB genomes.

**Additional file 22: Table S18.** Taxonomical classification of contigs in uncomplete MAGs based on CAT analysis.

**Additional file 23: Table S19.** Reference genomes.

## Data Availability

Raw sequences and metagenome-assembled genomes (MAGs) are available in NCBI under Bioproject numbers PRJNA507251 and PRJNA662092.
