# Supplementary material for: Single-colony sequencing reveals microbe-by-microbiome phylosymbiosis between the cyanobacterium Microcystis and its associated bacteria
Source: Microbiome. 2021 Sep 27;9:194. doi: 10.1186/s40168-021-01140-8 (PMC8477515; doi:10.1186/s40168-021-01140-8)
Supplement: Supplementary file 5 — Additional file 4. Supplementary figures. [file 40168_2021_1140_MOESM5_ESM.pdf]

Supplementary figure legends

Figure 1. ML Phylogenetic tree of *Microcystis* genomes (a) and ANIm clusters (b) corresponding to monophyletic clades with ANI values greater or equal to 99%.

Figure 2. Phylogenetic tree of *Microcystis* and 72 associated species (391 MAGs) recovered from *Microcystis* colonies. Taxonomic annotation for the genomes to different genera and species was done using Blastp of the RecA and RpoB proteins against the NCBI database and then complemented using the Genome Taxonomy Database Toolkit (GTDB-Tk) v1.0.2. The colored circles indicate the most prevalent genera in the *Microcystis* colony Microbiome.

Figure 3. Associated bacteria prevalence in the *Microcystis* colony microbiome from Canada and Brazil. Based on the normalized read coverages greater or equal than 1%, we recalculated the richness or number of associated species to the *Microcystis* colonies. The length of the whiskers represents 1.5 times of the interquartile range (IQR). The black line within each box represent the median. The lower and upper hinges represent the first and third quartiles.

Figure 4. Read cut-off values vs size of the core microbiome based on the Kaiju read counts and taxonomical annotation.

Figure 5. *Microcystis* genotype diversity over time in environmental metagenomes from Lake Champlain. Using MIDAS software, we estimated the relative abundances (a) and the read coverage (b) of the *Microcystis* genotypes in 72 shotgun metagenomes from Lake Champlain,

Quebec (62 metagenomes from a long-term experiment (2006 to 2016), plus 10 metagenomes from 2017 and 2018). The metagenomes with *Microcystis* genotype coverage lower than 1 or incomplete metadata were excluded.

Figure 6. *Microcystis* genotypic diversity variation over time. We used dbRDA (a) and NMDS (b) to ordinate the metagenome samples from Quebec based on the Bray–Curtis dissimilarities of the normalized coverage of *Microcystis* genotypes (NMDS stress value = 0.0859).

Figure 7. *Microcystis* genotype diversity over time in environmental metagenomes from Pampulha reservoir. The figure shows the relative abundances (a) and the read coverage (b). The genotypes containing the mcy cluster are indicated with an asterisk.

Figure 8. Associated bacteria (AB) diversity variation over time. We used dbRDA (a) and non-NMDS (b) to ordinate the metagenome samples from Quebec based on the Bray–Curtis dissimilarities of the normalized coverage of the AB species (NMDS stress value = 0.1569).

Figure 9. *Microcystis*, *Dolichospermum* and associated bacteria species coverage in environmental metagenomes across time. Figure 8a shows the *Microcystis* and *Dolichospermum* normalized read coverages across time. Figure 8b shows the *Microcystis*-associated bacteria genus read coverage.

Figure 10. Certain associated bacteria species are well correlated with the presence of *Microcystis*. Spearman correlation coefficients were estimated between *Microcystis* and each associated

bacterium and between *Dolichospermum* and each associated bacterium. Figure 9a shows the comparisons at genus level and Figure 9b the comparisons at species level. The axis x was sorted according with the genus and species prevalence in the *Microcystis* colonies from Canada. The correlations between the AB and *Microcystis* were also estimated using fastspar (Figure 9c and 9d).

Figure 11. *Microcystis* abundances are correlated with the nine most prevalent members of the microbiome in Lake Champlain. The scatter plots show the Spearman correlation between *Microcystis* and a particular AB or between *Dolichospermum* and a particular AB. The x and y axes show the log2 transformed read counts for *Microcystis* and each AB in the environmental metagenomes.

Figure 12. Scatter plot showing the relation between AB genera prevalence in the *Microcystis* colonies and the correlations values (Spearman and SparCC). The strongest correlated AB are those with the highest prevalence values.

Figure 13. *Microcystis* genotype-specific associations with associated bacteria species in environmental metagenomes. Normalized read counts were estimated per each *Microcystis* genotype and the associated bacteria species in 72 environmental metagenomes. The Spearman correlation coefficients were estimated between each *Microcystis* genotype and each associated bacteria species (Figure 12a). The correlations were also estimated using fastspar (Figure 12b).

Figure 14. PCA based on the presence absence of KEGG Orthologous genes in *Microcystis* and associated bacteria genera.

Figure 15. Number of colonies sequenced per each sampling point in Lake Champlain and Pampulha reservoir.

Figure 16. Pictures of the isolated and sequenced colonies.

Figure 17. Distribution of contigs sizes for each *Microcystis* colony metagenome assembly. The figure shows the kernel density estimations based on the contigs size in each metagenome assembly. Each colored line represents a metagenomic assembly. Noted that the individual assemblies generally contain an important number of contigs with sizes lower than 2,500 bp. The density estimations were plotted using the program ggplot2[1].

Figure 18. ML phylogenetic tree of *Microcystis* genomes without outgroups.

Figure 19. MIDAS workflow used to analyze the *Microcystis* colony and metagenome community composition.

Figure 20. Pearson correlation between *Microcystis* genotype coverage with alignment identity of ≥99% and *Microcystis* reference coverage with alignment identity of ≥96%.

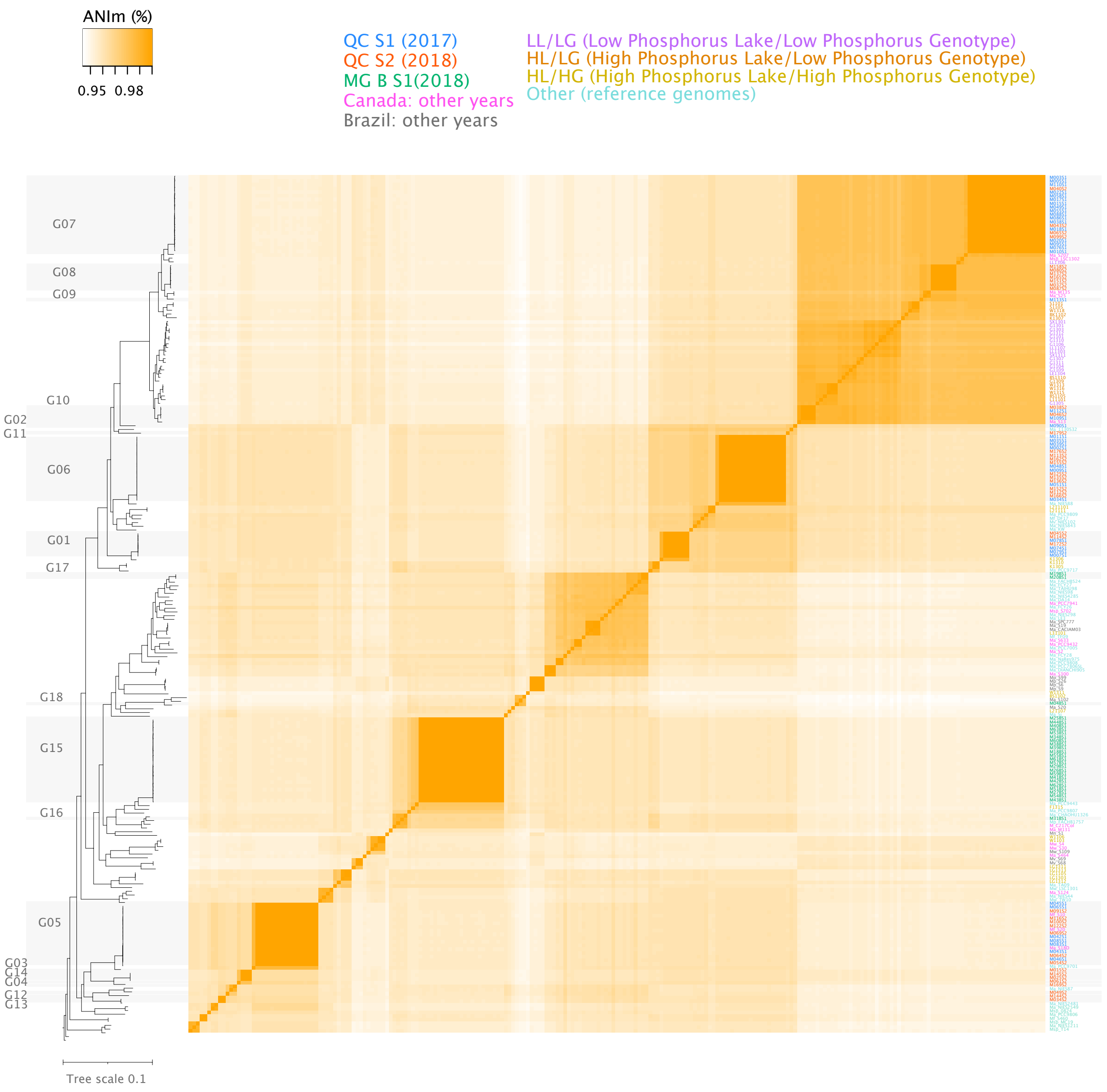
ANIm (%)
0.95 0.98
QC S1 (2017)
QC S2 (2018)
MG B S1(2018)
Canada: other years
Brazil: other years
LL/LG (Low Phosphorus Lake/Low Phosphorus Genotype)
HL/LG (High Phosphorus Lake/Low Phosphorus Genotype)
HL/HG (High Phosphorus Lake/High Phosphorus Genotype)
Other (reference genomes)
G07
G08
G09
G10
G02
G11
G06
G01
G17
G18
G15
G16
G05
G03
G14
G04
G12
G13
Tree scale 0.1

Fig. S1

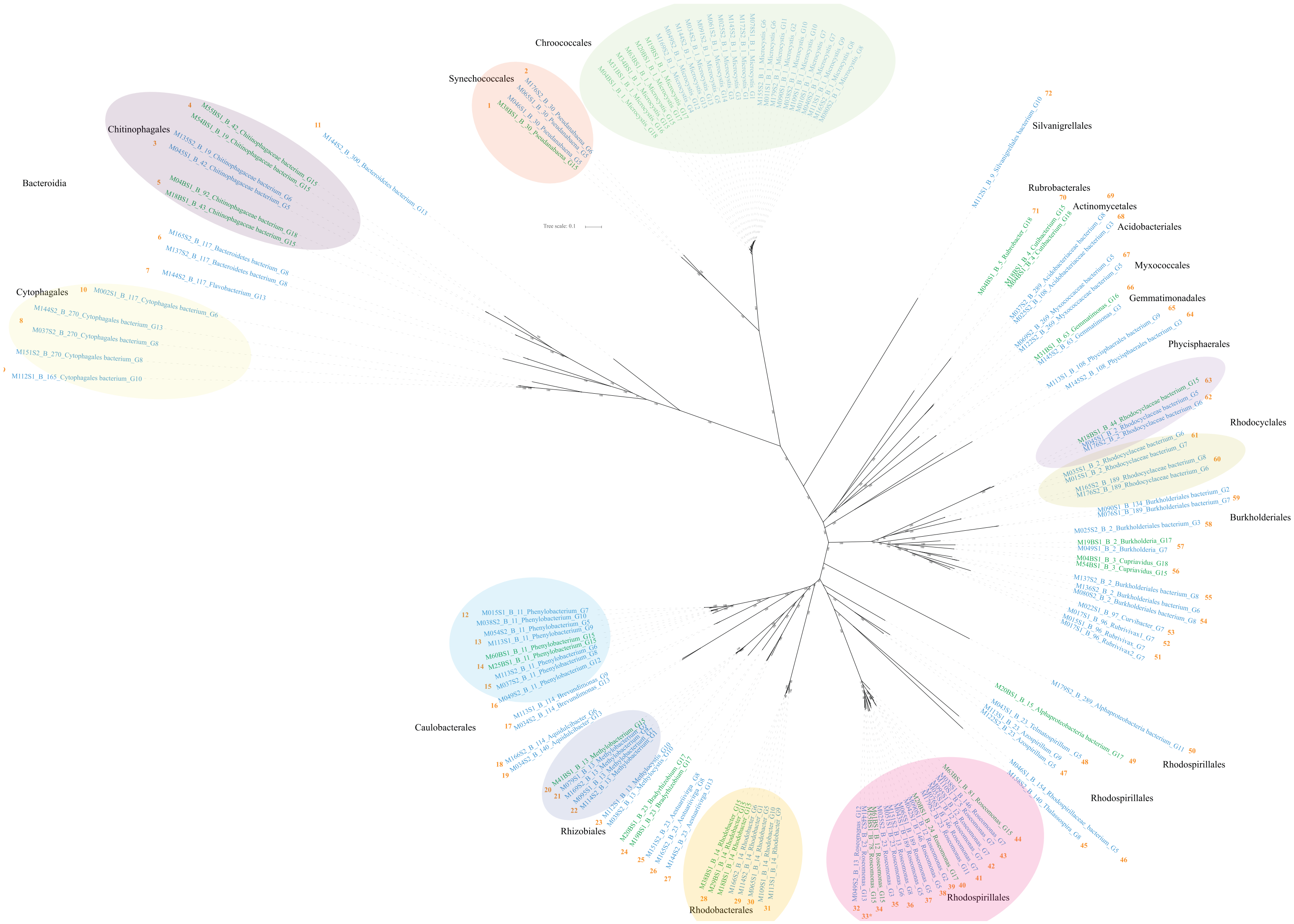
Chroococcales
Synechococcales
Chitinophagales
Bacteroidia
Cytophagales
Tree scale: 0.1
Silvanigrellales
Rubrobacterales
Actinomycetales
Acidobacteriales
Myxococcales
Gemmatimonadales
Phycisphaerales
Rhodocyclales
Burkholderiales
Rhodospirillales
Rhodospirillales
Caulobacterales
Rhizobiales
Rhodobacterales
Rhodospirillales

Fig. S2

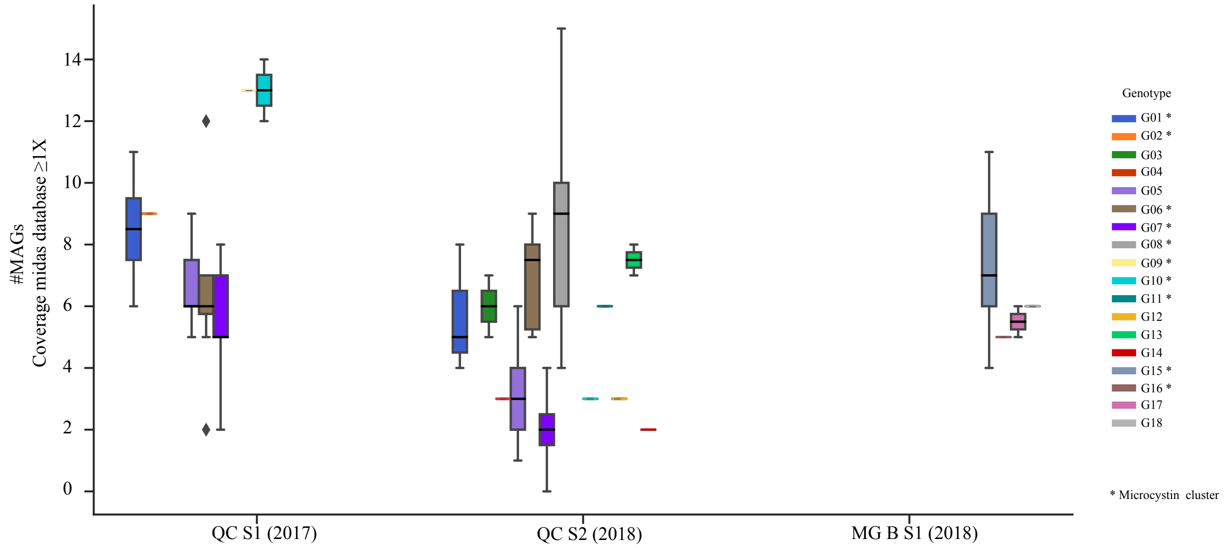
#MAGs
Coverage midas database ≥1X
14
12
10
8
6
4
2
0
QC S1 (2017)
QC S2 (2018)
MG B S1 (2018)
Genotype
G01 *
G02 *
G03
G04
G05
G06 *
G07 *
G08 *
G09 *
G10 *
G11 *
G12
G13
G14
G15 *
G16 *
G17
G18
* Microcystin cluster

Fig. S3

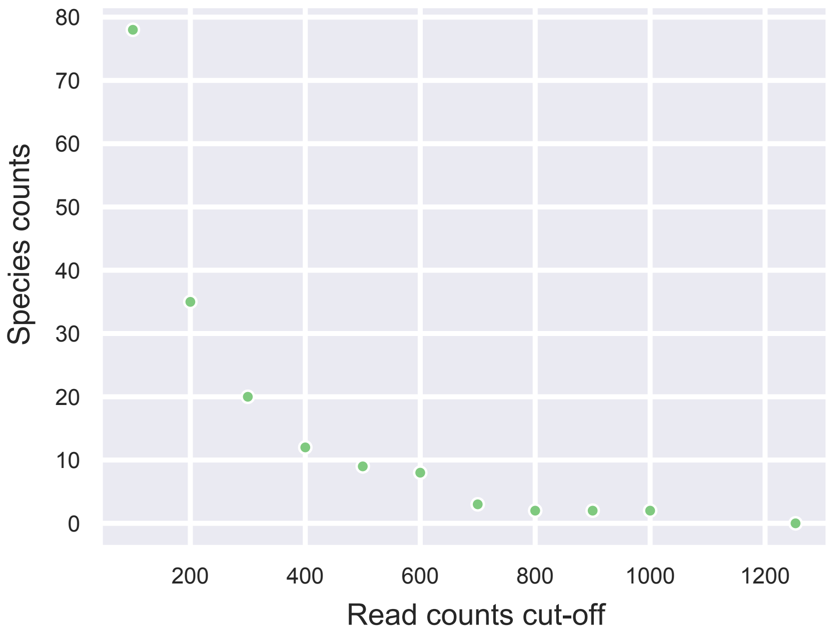
Species counts
80
70
60
50
40
30
20
10
0
200
400
600
800
1000
1200
Read counts cut-off

Fig. S4

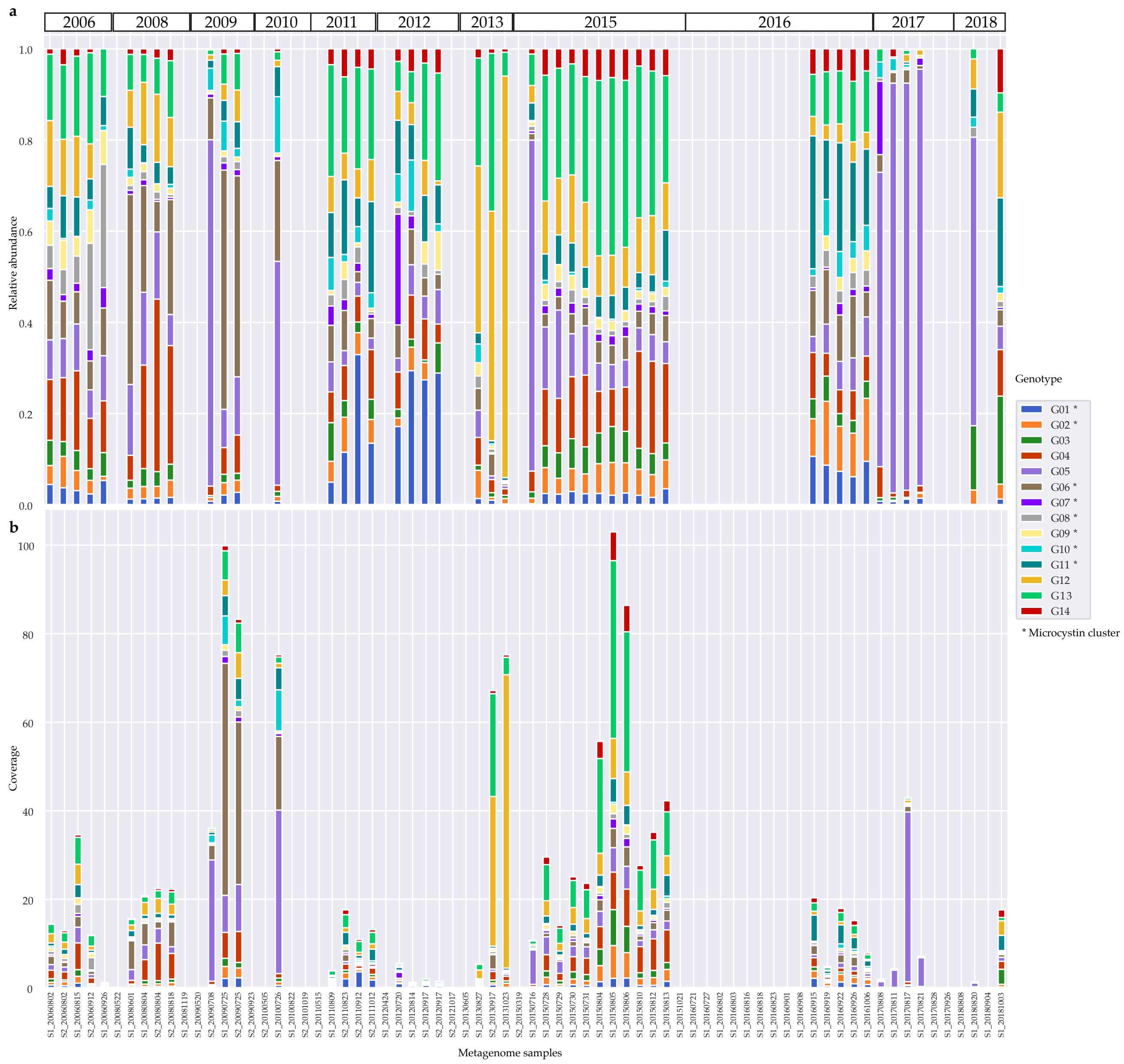
a
b
2006
2008
2009
2010
2011
2012
2013
2015
2016
2017
2018
Relative abundance
Coverage
Metagenome samples
Genotype
G01 *
G02 *
G03
G04
G05
G06 *
G07 *
G08 *
G09 *
G10 *
G11 *
G12
G13
G14
* Microcystin cluster

Fig. S5

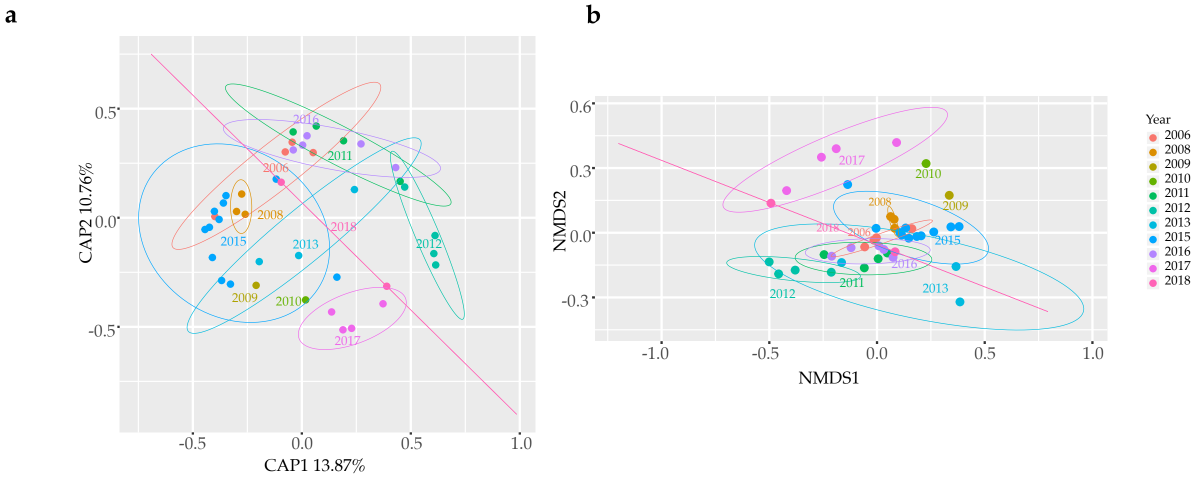
a
b
CAP2 10.76%
CAP1 13.87%
NMDS2
NMDS1
Year
2006
2008
2009
2010
2011
2012
2013
2015
2016
2017
2018

Fig. S6

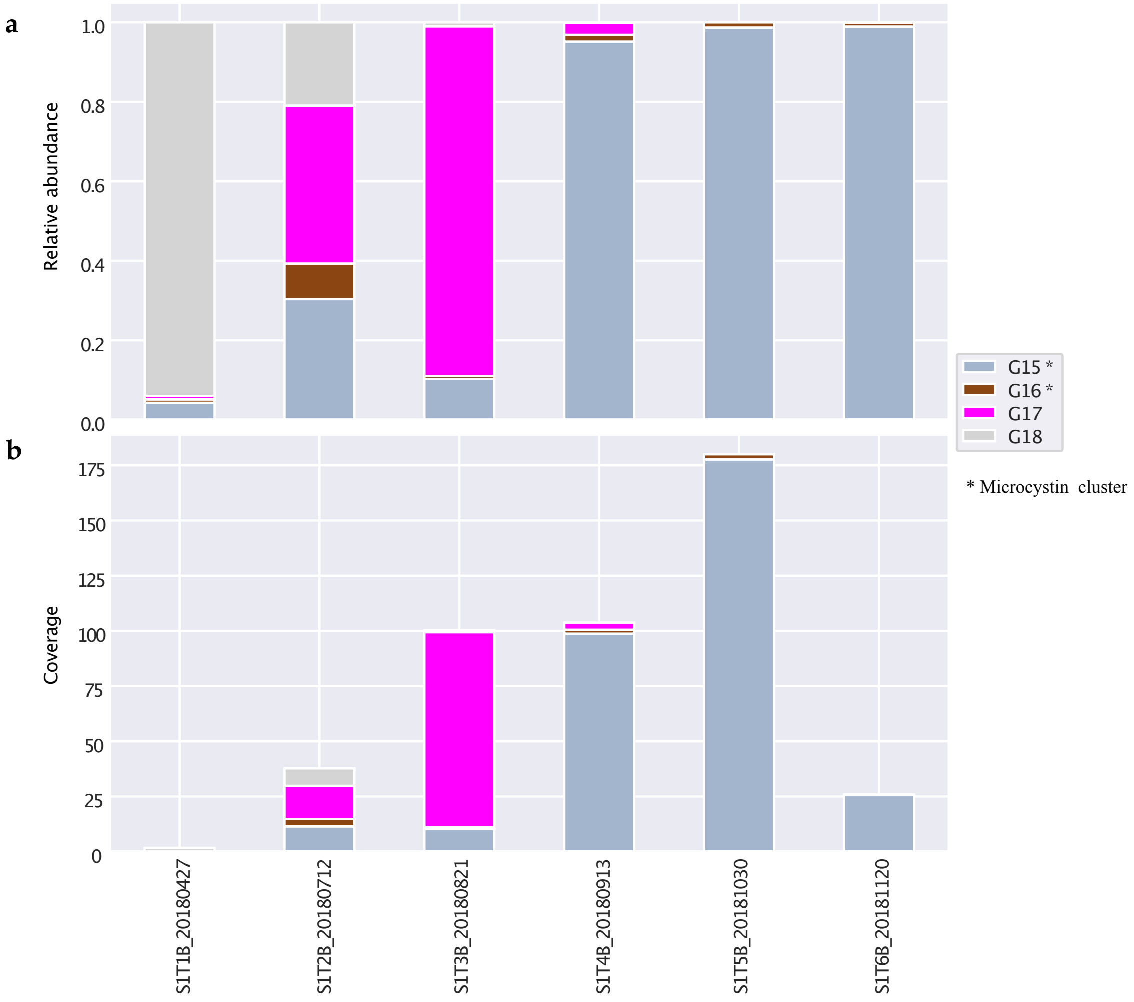
a
b
Relative abundance
Coverage
G15 *
G16 *
G17
G18
* Microcystin cluster
S1T1B_20180427
S1T2B_20180712
S1T3B_20180821
S1T4B_20180913
S1T5B_20181030
S1T6B_20181120

Fig. S7

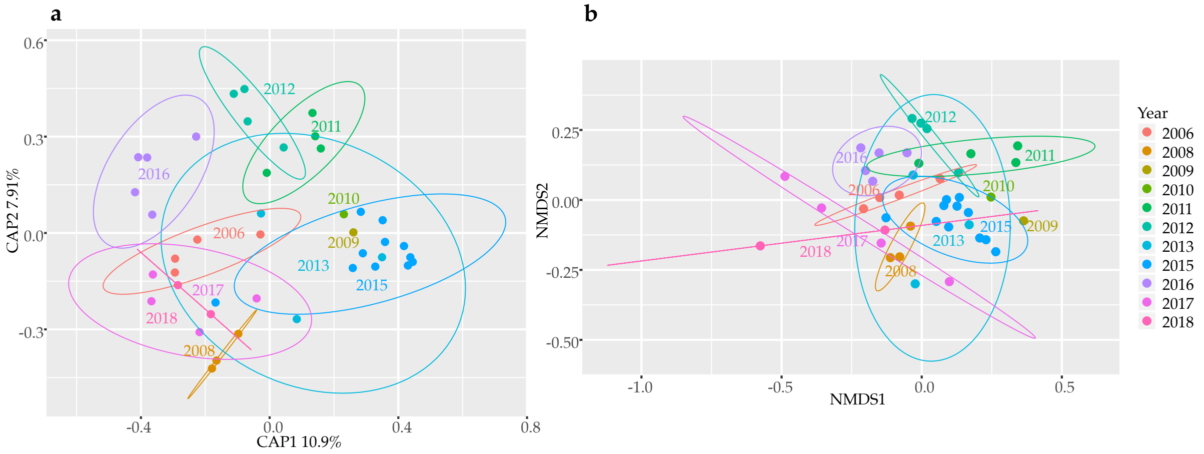
a
b
CAP2 7.91%
CAP1 10.9%
0.6
0.3
0.0
-0.3
-0.4
0.0
0.4
0.8
NMDS2
NMDS1
0.25
0.00
-0.25
-0.50
-1.0
-0.5
0.0
0.5
2006
2008
2009
2010
2011
2012
2013
2015
2016
2017
2018
Year

Fig. S8

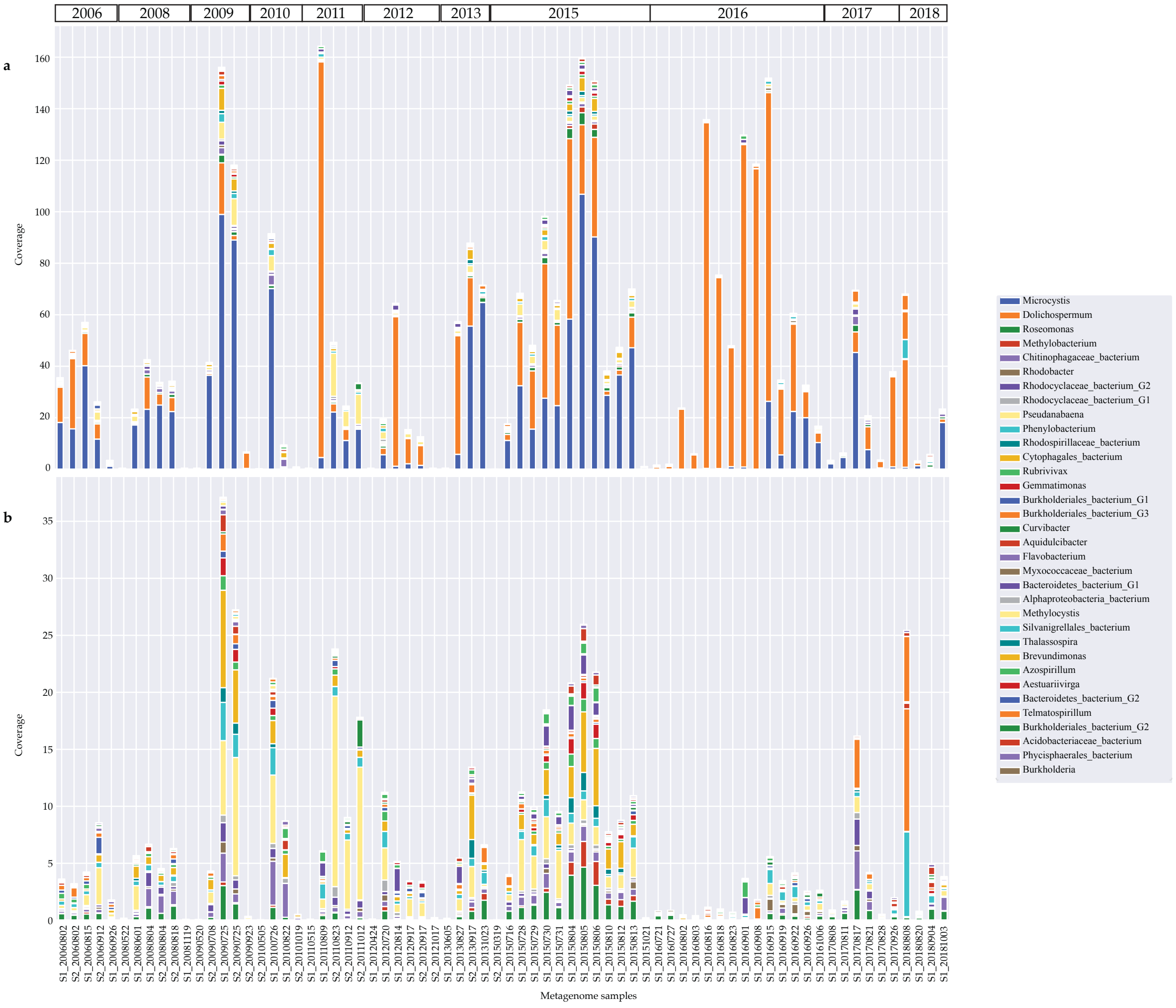
2006
2008
2009
2010
2011
2012
2013
2015
2016
2017
2018
a
b
Coverage
Metagenome samples
Microcystis
Dolichospermum
Roseomonas
Methylobacterium
Chitinophagaceae_bacterium
Rhodobacter
Rhodocyclaceae_bacterium_G2
Rhodocyclaceae_bacterium_G1
Pseudanabaena
Phenylobacterium
Rhodospirillaceae_bacterium
Cytophagales_bacterium
Rubrivivax
Gemmatimonas
Burkholderiales_bacterium_G1
Burkholderiales_bacterium_G3
Curvibacter
Aquiduleibacter
Flavobacterium
Myxococcaceae_bacterium
Bacteroidetes_bacterium_G1
Alphaproteobacteria_bacterium
Methylocystis
Silvanigrellales_bacterium
Thalassospira
Brevundimonas
Azospirillum
Aestuariivirga
Bacteroidetes_bacterium_G2
Telmatospirillum
Burkholderiales_bacterium_G2
Acidobacteriaceae_bacterium
Phycisphaerales_bacterium
Burkholderia

Fig. S9

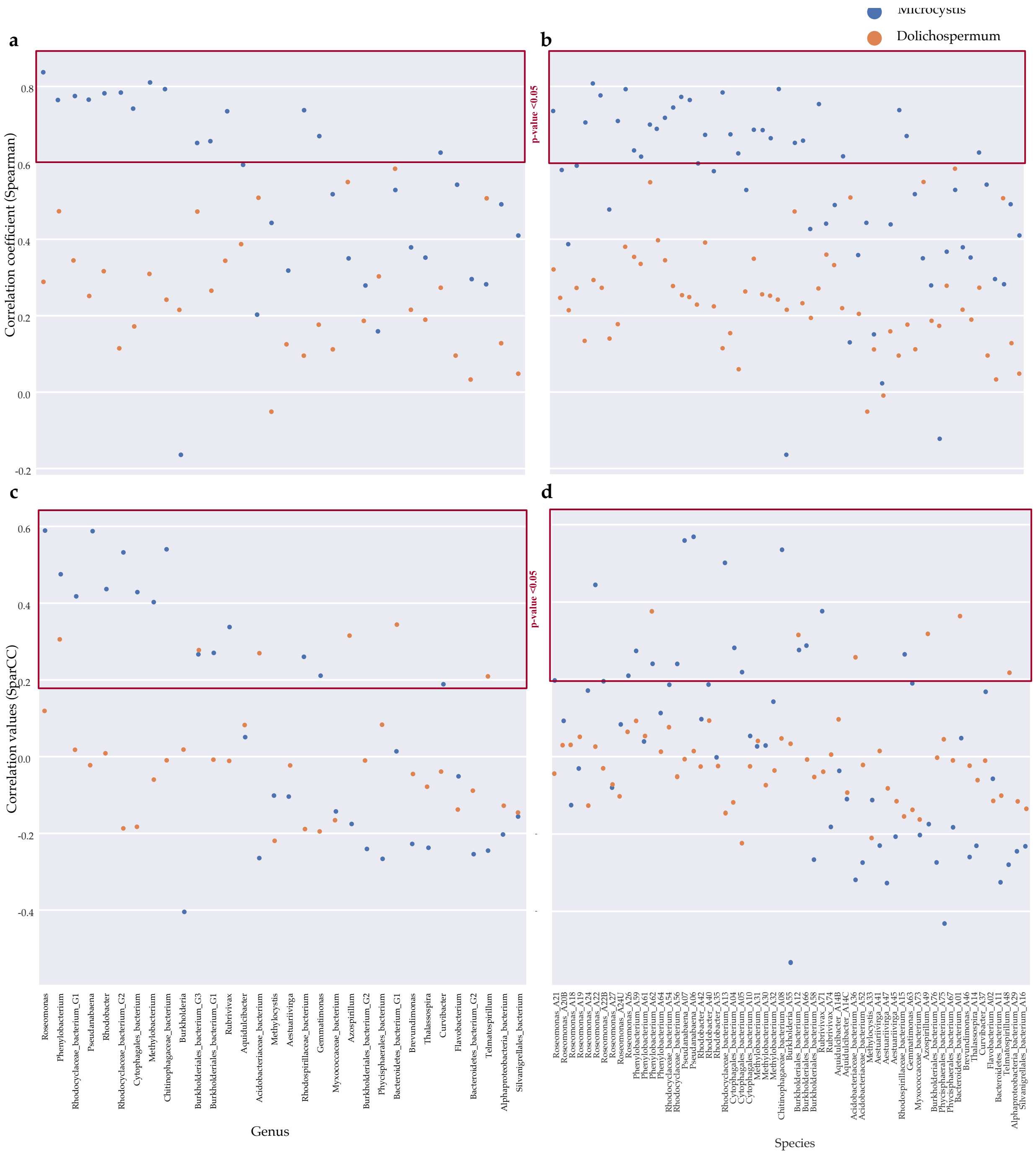
a
b
c
d
Microcystis
Dolichospermum
Correlation coefficient (Spearman)
Correlation values (SparCC)
p-value <0.05
p-value <0.05
Genus
Species
Roseomonas
Phenylobacterium
Rhodocyclaceae_bacterium_G1
Pseudanabaena
Rhodobacter
Rhodocyclaceae_bacterium_G2
Cytophagales_bacterium
Methylobacterium
Chitinophagaceae_bacterium
Burkholderia
Burkholderiales_bacterium_G3
Burkholderiales_bacterium_G1
Rubrivivax
Aquidulcibacter
Acidobacteriaceae_bacterium
Methylocystis
Aestuariivirga
Rhodospirillaceae_bacterium
Gemmatimonas
Myxococcaceae_bacterium
Azospirillum
Burkholderiales_bacterium_G2
Phycisphaerales_bacterium
Bacteroidetes_bacterium_G1
Brevundimonas
Thalassospira
Curvibacter
Flavobacterium
Bacteroidetes_bacterium_G2
Telmatospirillum
Alphaproteobacteria_bacterium
Silvanigrellales_bacterium

Fig. S10

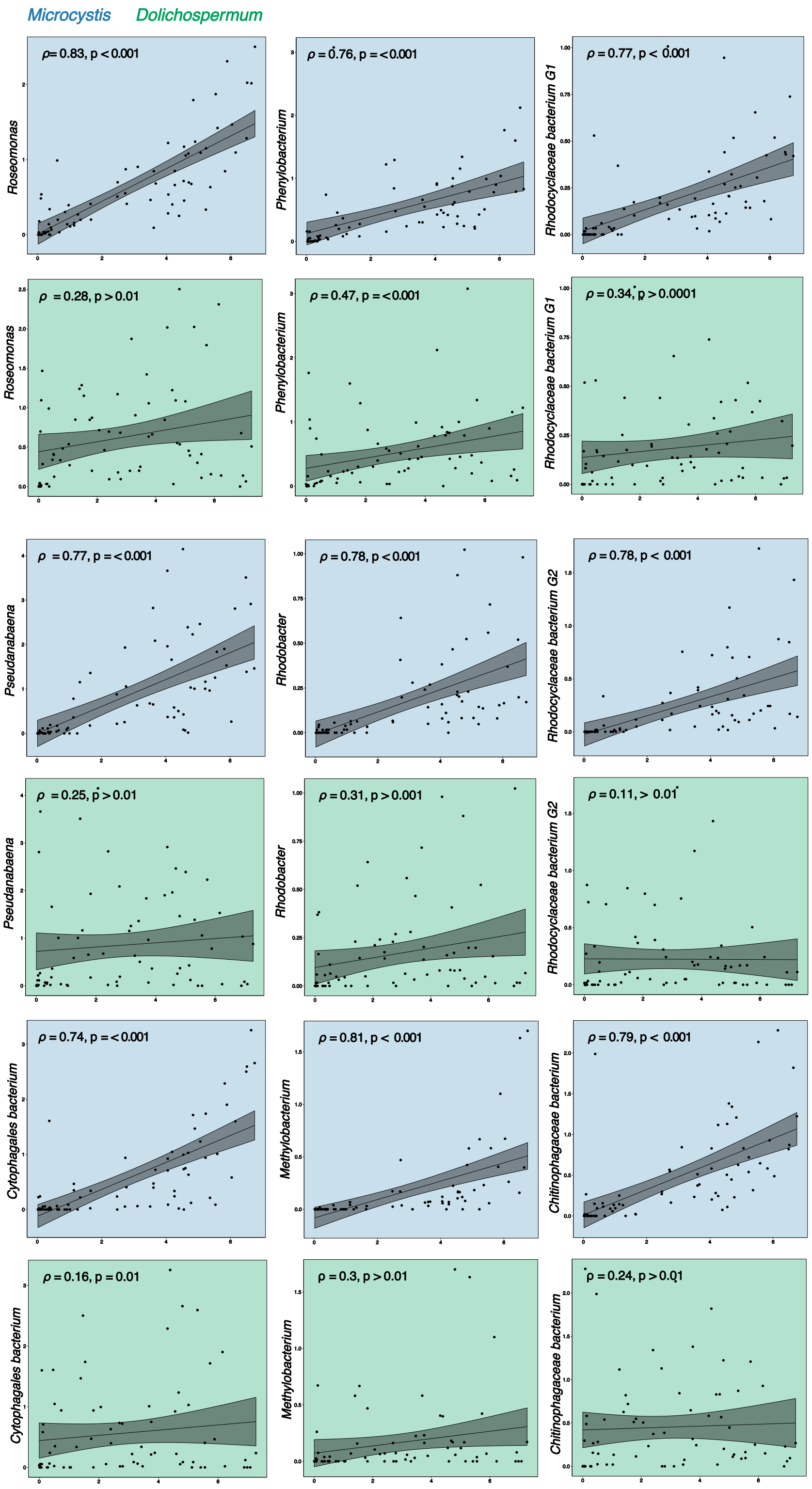
Microcystis
Dolichospermum
ρ= 0.83, p < 0.001
Roseomonas
ρ = 0.76, p = < 0.001
Phenylobacterium
ρ = 0.77, p < 0.001
Rhodocyclaceae bacterium G1
ρ = 0.28, p > 0.01
Roseomonas
ρ = 0.47, p = < 0.001
Phenylobacterium
ρ = 0.34, p > 0.0001
Rhodocyclaceae bacterium G1
ρ = 0.77, p = < 0.001
Pseudanabaena
ρ = 0.78, p < 0.001
Rhodobacter
ρ = 0.78, p < 0.001
Rhodocyclaceae bacterium G2
ρ = 0.25, p > 0.01
Pseudanabaena
ρ = 0.31, p > 0.001
Rhodobacter
ρ = 0.11, > 0.01
Rhodocyclaceae bacterium G2
ρ = 0.74, p = < 0.001
Cytophagales bacterium
ρ = 0.81, p < 0.001
Methylobacterium
ρ = 0.79, p < 0.001
Chitinophagaceae bacterium
ρ = 0.16, p = 0.01
Cytophagales bacterium
ρ = 0.3, p > 0.01
Methylobacterium
ρ = 0.24, p > 0.01
Chitinophagaceae bacterium

Fig. S11

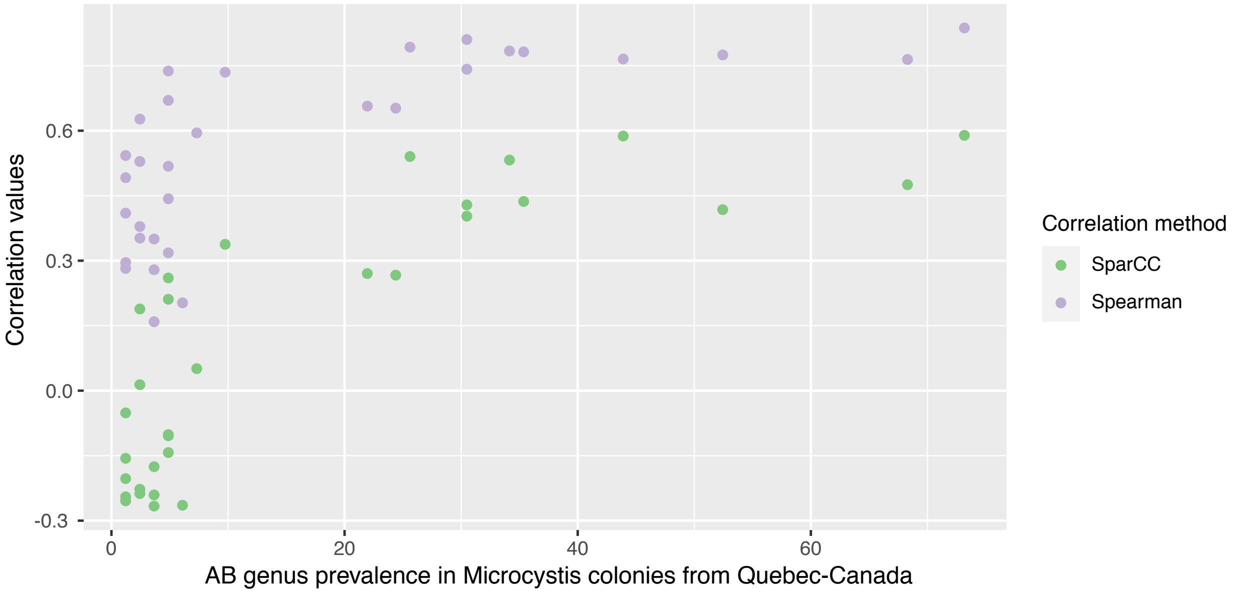

Correlation values
0.6
0.3
0.0
-0.3
0
20
40
60
AB genus prevalence in Microcystis colonies from Quebec-Canada
Correlation method
SparCC
Spearman

Fig. S12

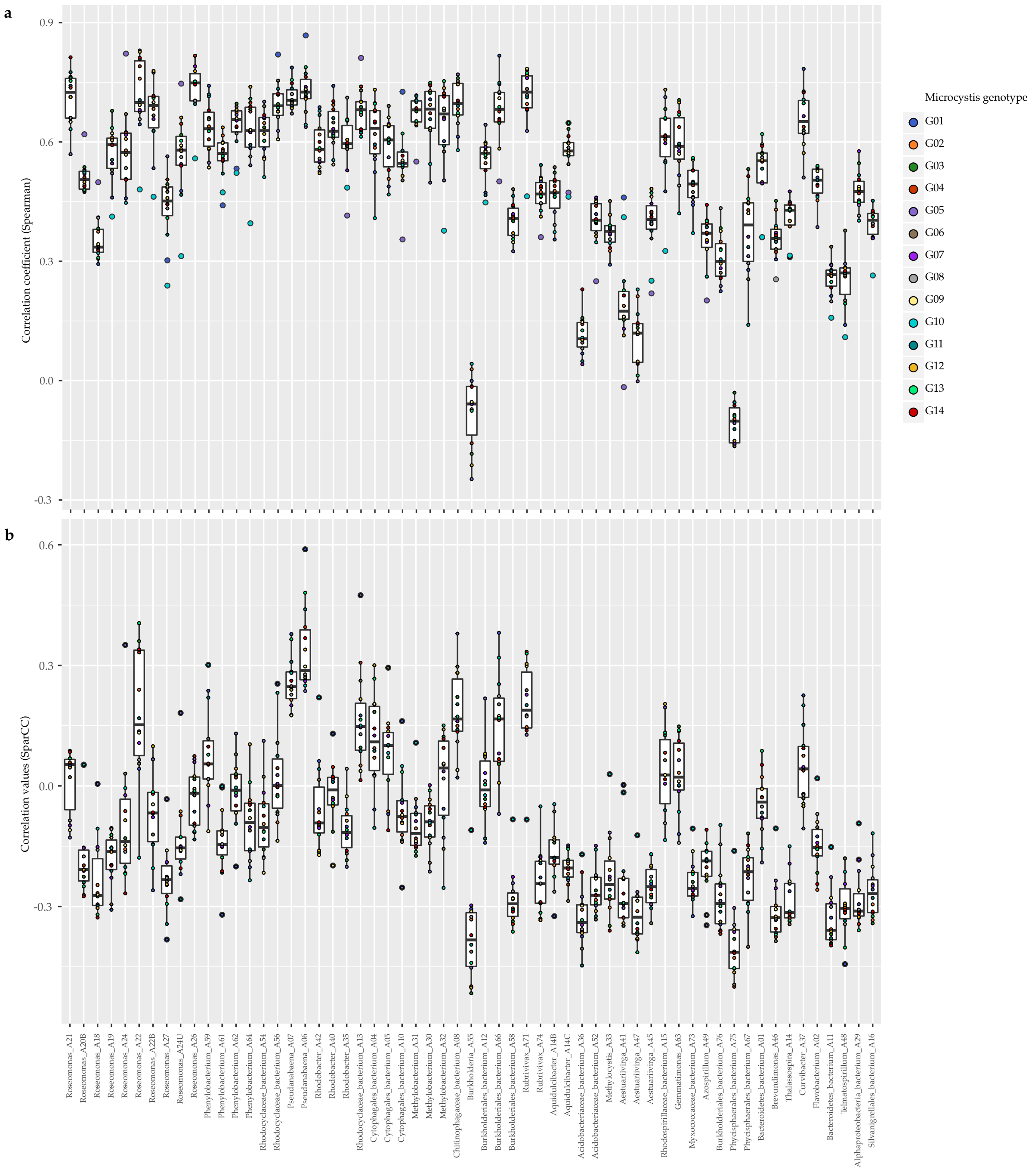

a
b
Correlation coefficient (Spearman)
Correlation values (SparCC)
0.9
0.6
0.3
0.0
-0.3
Microcystis genotype
G01
G02
G03
G04
G05
G06
G07
G08
G09
G10
G11
G12
G13
G14
Roseomonas_A21
Roseomonas_A20B
Roseomonas_A18
Roseomonas_A19
Roseomonas_A24
Roseomonas_A22
Roseomonas_A22B
Roseomonas_A27
Roseomonas_A24U
Roseomonas_A26
Phenylobacterium_A59
Phenylobacterium_A61
Phenylobacterium_A62
Phenylobacterium_A64
Rhodocyclaceae_bacterium_A54
Rhodocyclaceae_bacterium_A56
Pseudanabaena_A07
Pseudanabaena_A06
Rhodobacter_A42
Rhodobacter_A40
Rhodobacter_A35
Rhodocyclaceae_bacterium_A13
Cytophagales_bacterium_A04
Cytophagales_bacterium_A05
Cytophagales_bacterium_A10
Methylobacterium_A31
Methylobacterium_A30
Methylobacterium_A32
Chitinophagaceae_bacterium_A08
Burkholderia_A55
Burkholderiales_bacterium_A12
Burkholderiales_bacterium_A66
Burkholderiales_bacterium_A58
Rubrivivax_A71
Rubrivivax_A74
Aquidulcibacter_A14B
Aquidulcibacter_A14C
Acidobacteriaceae_bacterium_A36
Acidobacteriaceae_bacterium_A52
Methylocystis_A33
Aestuariivirga_A41
Aestuariivirga_A47
Aestuariivirga_A45
Rhodospirillaceae_bacterium_A15
Gemmatimonas_A63
Myxococcaceae_bacterium_A73
Azospirillum_A49
Burkholderiales_bacterium_A76
Phycisphaerales_bacterium_A75
Phycisphaerales_bacterium_A67
Bacteroidetes_bacterium_A01
Brevundimonas_A46
Thalassospira_A14
Curvibacter_A37
Flavobacterium_A02
Bacteroidetes_bacterium_A11
Telmatospirillum_A48
Alphaproteobacteria_bacterium_A29
Silvanigrellales_bacterium_A16
Species

Fig. S13

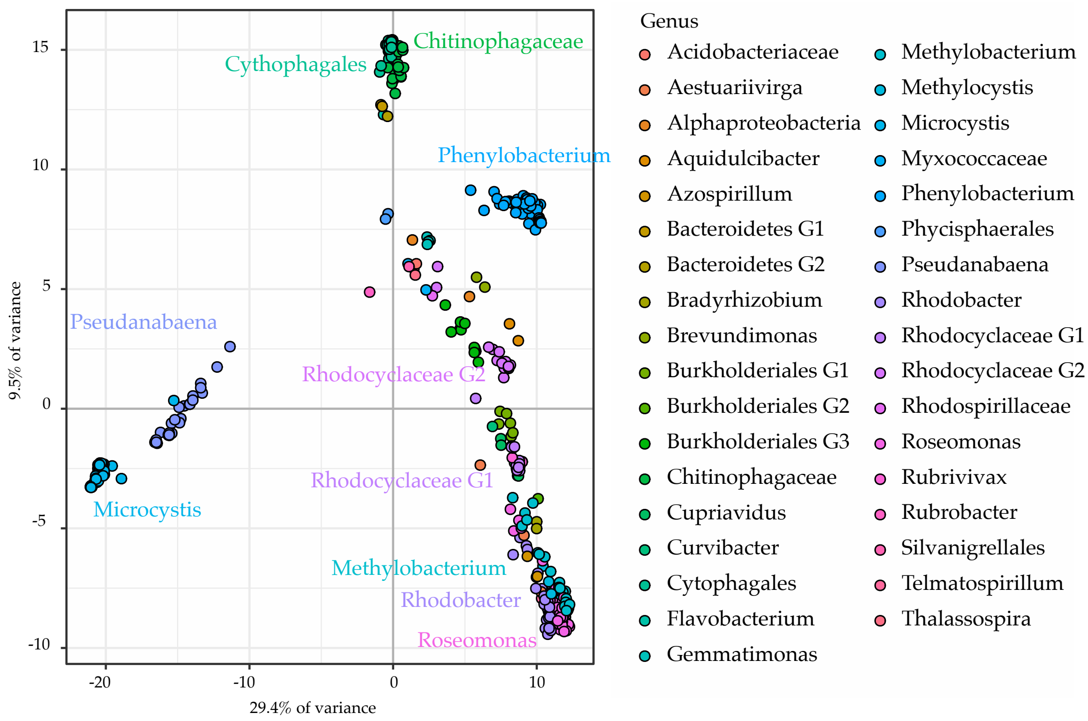

Fig. S14

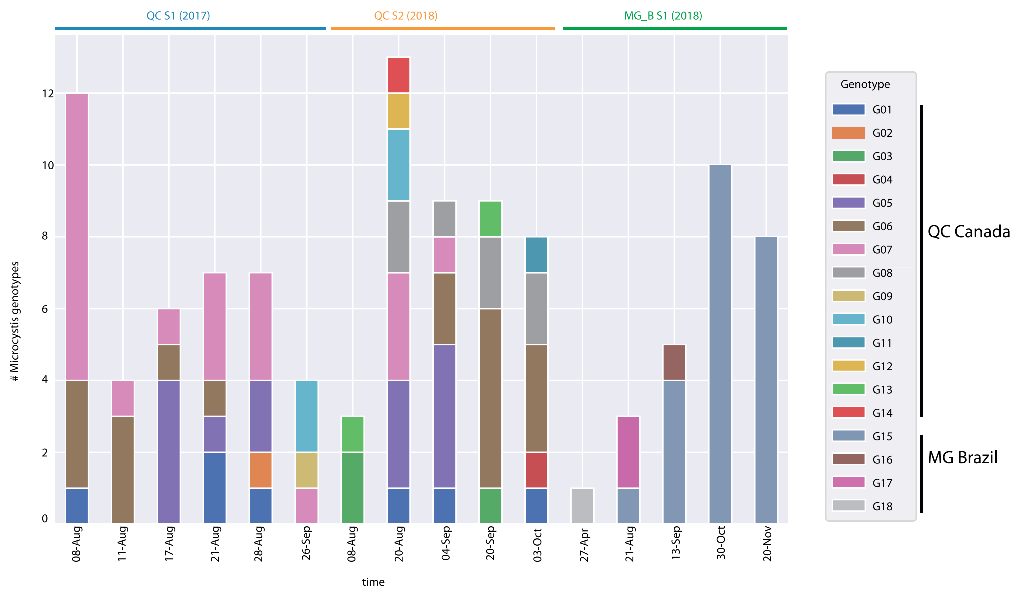

Fig. S15

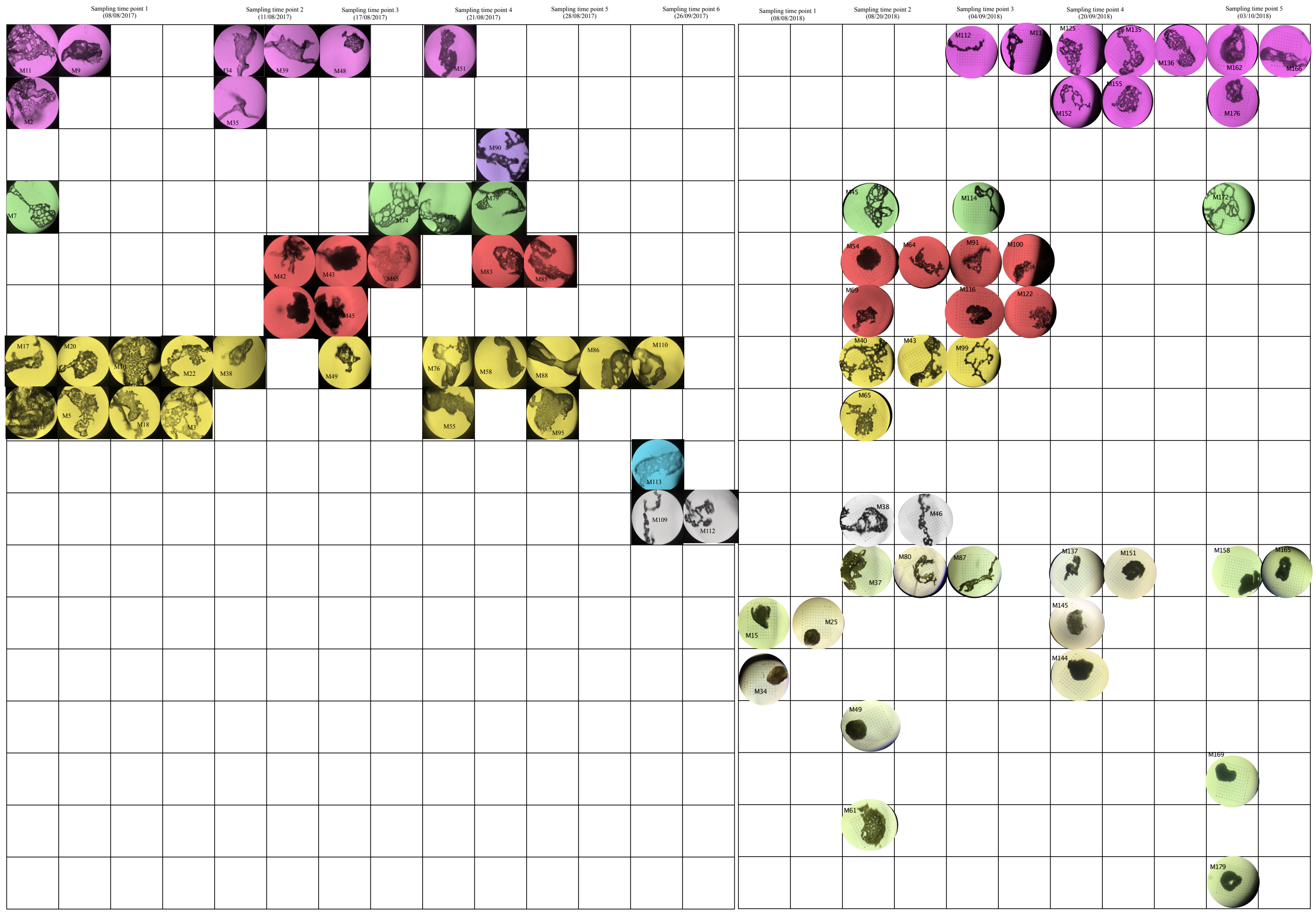
Sampling time point 1
(08/08/2017)
Sampling time point 2
(11/08/2017)
Sampling time point 3
(17/08/2017)
Sampling time point 4
(21/08/2017)
Sampling time point 5
(28/08/2017)
Sampling time point 6
(26/09/2017)
Sampling time point 1
(08/08/2018)
Sampling time point 2
(08/20/2018)
Sampling time point 3
(04/09/2018)
Sampling time point 4
(20/09/2018)
Sampling time point 5
(03/10/2018)

Fig. S16

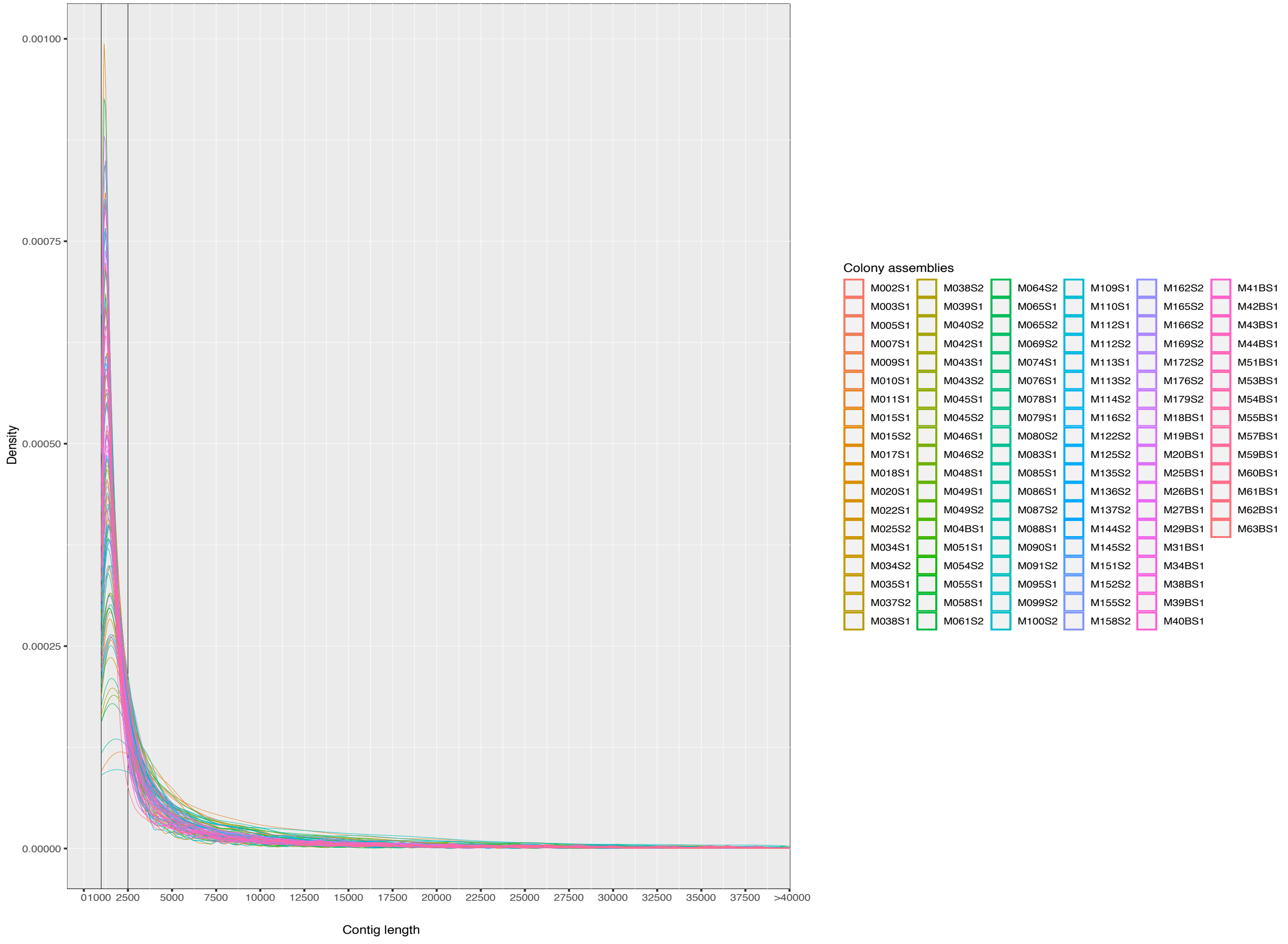
Density
0.00100
0.00075
0.00050
0.00025
0.00000
01000 2500
5000
7500
10000
12500
15000
17500
20000
22500
25000
27500
30000
32500
35000
37500
>40000
Contig length
Colony assemblies
M002S1
M003S1
M005S1
M007S1
M009S1
M010S1
M011S1
M015S1
M015S2
M017S1
M018S1
M020S1
M022S1
M025S2
M034S1
M034S2
M035S1
M037S2
M038S1
M038S2
M039S1
M040S2
M042S1
M043S1
M043S2
M045S1
M045S2
M046S1
M046S2
M048S1
M049S1
M049S2
M04BS1
M051S1
M054S2
M055S1
M058S1
M061S2
M064S2
M065S1
M065S2
M069S2
M074S1
M076S1
M078S1
M079S1
M080S2
M083S1
M085S1
M086S1
M087S2
M088S1
M090S1
M091S2
M095S1
M099S2
M100S2
M109S1
M110S1
M112S1
M112S2
M113S1
M113S2
M114S2
M116S2
M122S2
M125S2
M135S2
M136S2
M137S2
M144S2
M145S2
M151S2
M152S2
M155S2
M158S2
M162S2
M165S2
M166S2
M169S2
M172S2
M176S2
M179S2
M18BS1
M19BS1
M20BS1
M25BS1
M26BS1
M27BS1
M29BS1
M31BS1
M34BS1
M38BS1
M39BS1
M40BS1
M41BS1
M42BS1
M43BS1
M44BS1
M51BS1
M53BS1
M54BS1
M55BS1
M57BS1
M59BS1
M60BS1
M61BS1
M62BS1
M63BS1

Fig. S17

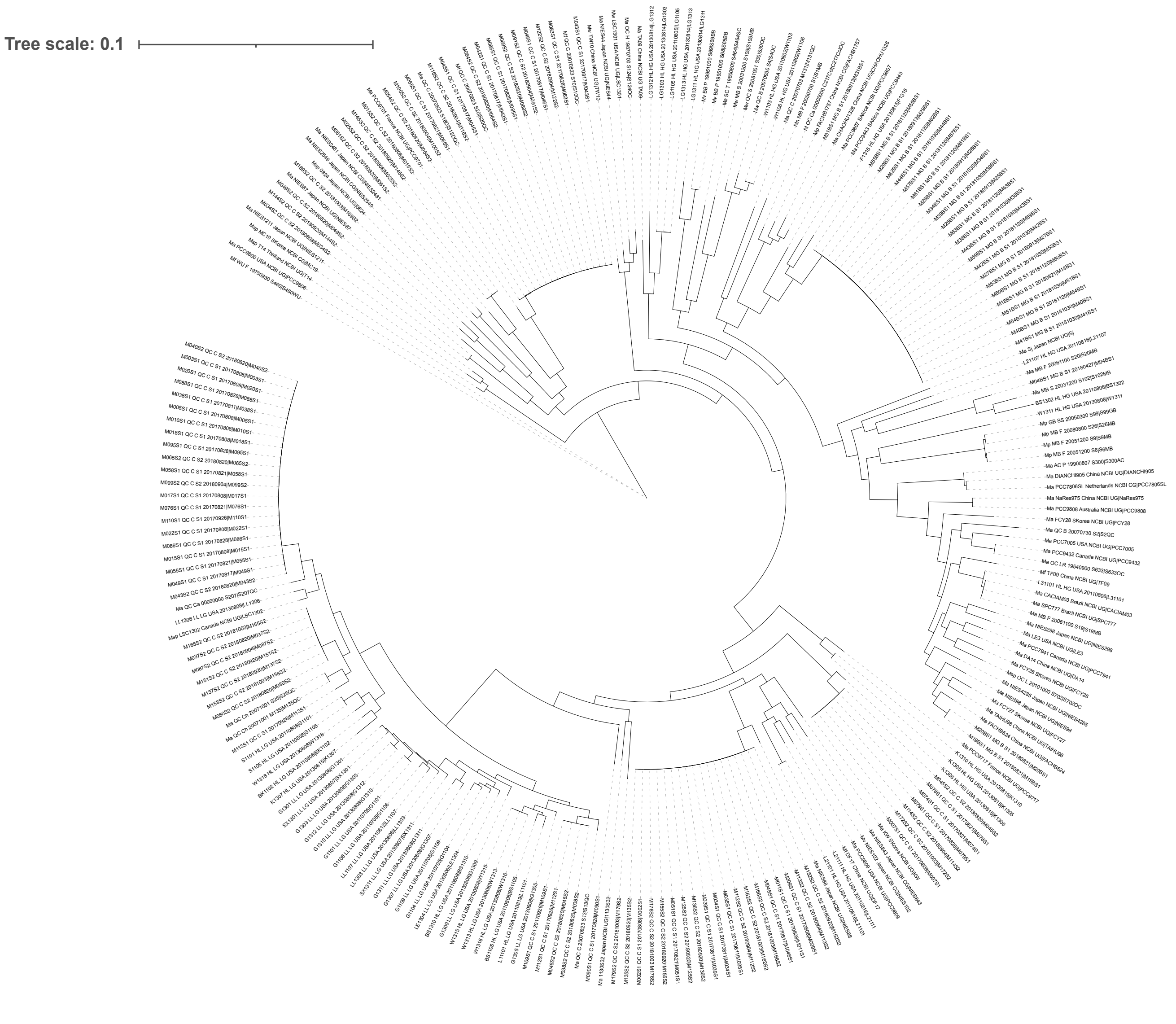

Fig. S18

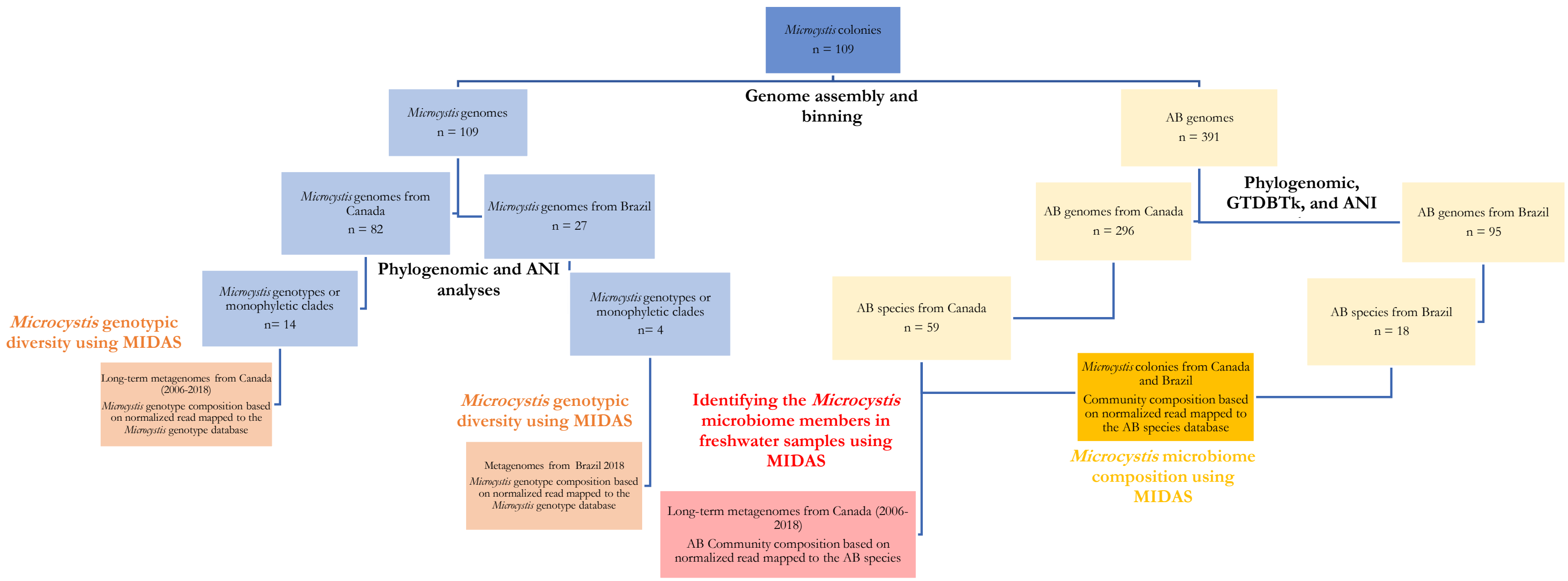

Microcystis colonies
n = 109
Genome assembly and binning
Microcystis genomes
n = 109
AB genomes
n = 391
Microcystis genomes from Canada
n = 82
Microcystis genomes from Brazil
n = 27
Phylogenomic and ANI analyses
Phylogenomic, GTDBTk, and ANI
AB genomes from Canada
n = 296
AB genomes from Brazil
n = 95
Microcystis genotypes or monophyletic clades
n= 14
Microcystis genotypes or monophyletic clades
n= 4
AB species from Canada
n = 59
AB species from Brazil
n = 18
Microcystis genotypic diversity using MIDAS
Long-term metagenomes from Canada (2006-2018)
Microcystis genotype composition based on normalized read mapped to the Microcystis genotype database
Microcystis genotypic diversity using MIDAS
Metagenomes from Brazil 2018
Microcystis genotype composition based on normalized read mapped to the Microcystis genotype database
Identifying the Microcystis microbiome members in freshwater samples using MIDAS
Long-term metagenomes from Canada (2006-2018)
AB Community composition based on normalized read mapped to the AB species
Microcystis colonies from Canada and Brazil
Community composition based on normalized read mapped to the AB species database
Microcystis microbiome composition using MIDAS

Fig. S19

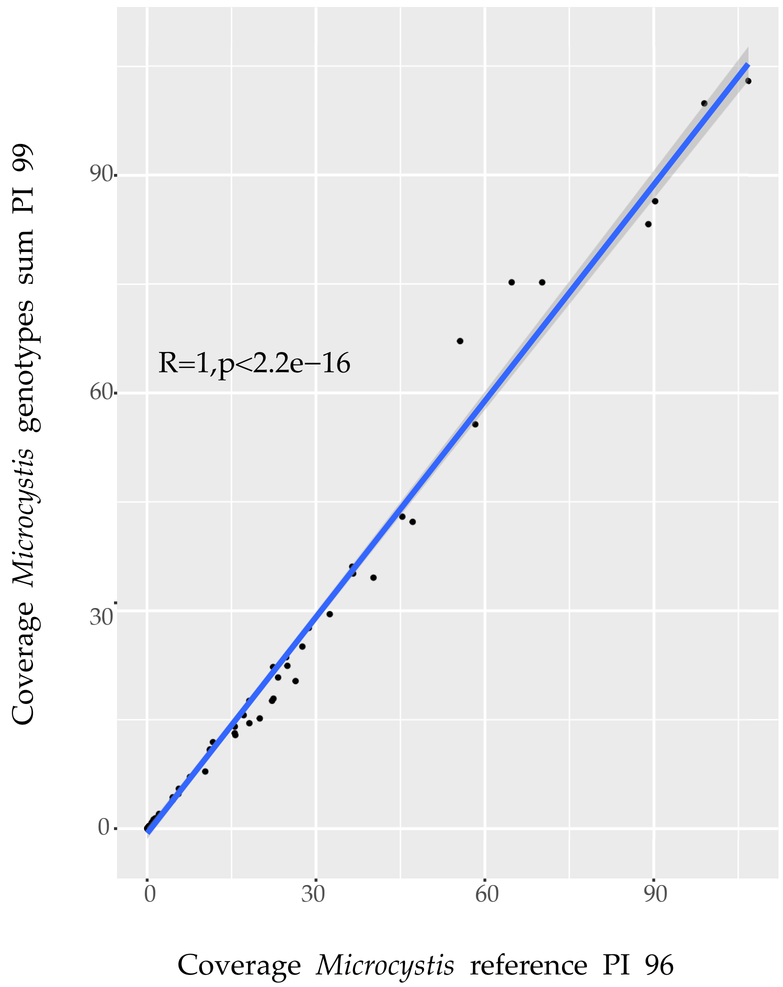
R=1,p<2.2e−16
Coverage *Microcystis* genotypes sum PI 99
Coverage *Microcystis* reference PI 96
0
30
60
90

Fig. S20

References:

1. Villanueva RAM, Chen ZJ. ggplot2: Elegant Graphics for Data Analysis, 2nd edition. Meas-Interdiscip Res. 2019;17:160-7.
